# There Is a Future for *N*-Heterocyclic Carbene Iron(II) Dyes in Dye-Sensitized Solar Cells: Improving Performance through Changes in the Electrolyte

**DOI:** 10.3390/ma12244181

**Published:** 2019-12-12

**Authors:** Mariia Karpacheva, Vanessa Wyss, Catherine E. Housecroft, Edwin C. Constable

**Affiliations:** Department of Chemistry, University of Basel, BPR 1096, Mattenstrasse 24a, CH-4058 Basel, Switzerland; mariia.karpacheva@unibas.ch (M.K.); vanessa.wyss@unibas.ch (V.W.); catherine.housecroft@unibas.ch (C.E.H.)

**Keywords:** iron, *N*-heterocyclic carbene, dye-sensitized solar cell, electrolyte, ionic liquid, lithium ion

## Abstract

By systematic tuning of the components of the electrolyte, the performances of dye-sensitized solar cells (DSCs) with an *N*-heterocyclic carbene iron(II) dye have been significantly improved. The beneficial effects of an increased Li^+^ ion concentration in the electrolyte lead to photoconversion efficiencies (PCEs) up to 0.66% for fully masked cells (representing 11.8% relative to 100% set for N719) and an external quantum efficiency maximum (EQE_max_) up to approximately 25% due to an increased short-circuit current density (*J*_SC_). A study of the effects of varying the length of the alkyl chain in 1-alkyl-3-methylimidazolium iodide ionic liquids (ILs) shows that a longer chain results in an increase in *J*_SC_ with an overall efficiency up to 0.61% (10.9% relative to N719 set at 100%) on going from *n*-methyl to *n*-butyl chain, although an *n*-hexyl chain leads to no further gain in PCE. The results of electrochemical impedance spectroscopy (EIS) support the trends in *J*_SC_ and open-circuit voltage (*V*_OC_) parameters. A change in the counterion from I^−^ to [BF_4_]^−^ for 1-propyl-3-methylimidazolium iodide ionic liquid leads to DSCs with a remarkably high *J*_SC_ value for an *N*-heterocyclic carbene iron(II) dye of 4.90 mA cm^−2^, but a low *V*_OC_ of 244 mV. Our investigations have shown that an increased concentration of Li^+^ in combination with an optimized alkyl chain length in the 1-alkyl-3-methylimidazolium iodide IL in the electrolyte leads to iron(II)-sensitized DSC performances comparable with those of containing some copper(I)-based dyes.

## 1. Introduction

Over the past several decades, solar cells have evolved as a key technology in the field of renewable energy [1]. Solar cells convert solar into electrical energy. Today, most of the commercially available solar cells are made of crystalline silicon (c-Si) [2]. However, their tedious and costly fabrication or the necessity of using environmentally non-benign metals such as gallium or cadmium as alternative semiconductors are major drawbacks for their use as a green and sustainable energy source [3]. Dye-sensitized solar cells (DSCs) represent an alternative to overcome these limitations [4]. Lower material costs and less sophisticated manufacturing processes combined with the avoidance of toxic materials offer considerable advantages [5], and the upscaling from a research laboratory to commercial DSCs makes their introduction to the market viable [5,6,7].

**Figure 1:**
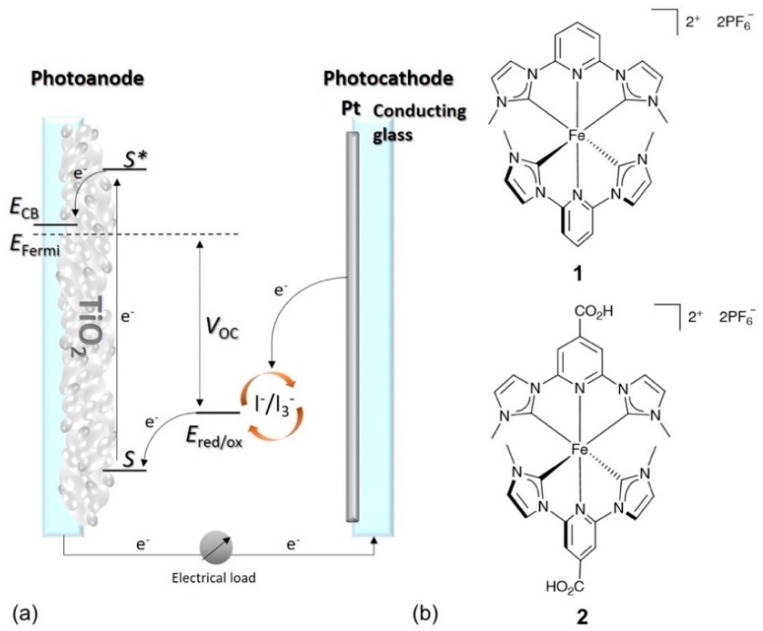
(**a**) Composition of an n-type DSC. Arrows show the direction of the electrical circuit. *V*_OC_ = open circuit voltage, which is the maximum potential that the DSC can provide. (**b**) Structures of the iron (II) NHC complexes **1** and **2**.

DSCs consist of a photoanode, an electrolyte containing a redox shuttle, and a photocathode (Figure 1a). Photoexcitation of the dye (S→S*) results in electron injection into the conduction band (CB) of the semiconductor, which is typically mesoporous TiO_2_ in an n-type DSC. The electron travels through the electrical load and, at the platinum-coated counter electrode, it reduces a redox couple, typically consisting of iodide/triiodide (I^−^/I_3_^−^). The reduced form of the redox couple regenerates the dye in the ground state, which completes the circuit [8].

Since every part of the cell contributes to the overall performance of a DSC, optimization of semiconductors [9,10,11,12,13], sensitizers [14,15,16,17,18,19,20], counter electrodes [21,22,23], and electrolytes [16,24,25,26,27,28,29,30,31,32] are all critical. The most widely used sensitizers with photoconversion efficiencies (PCE) of up to 11% are ruthenium-oligopyridine complexes [16,33,34,35]. Upon absorption of light, these dyes can provide an efficient metal-to-ligand charge transfer (MLCT) with a long lifetime and a low-energy excited state. This results in efficient electron injection into the semiconductor.

However, the use of metals with low natural abundancies significantly increases the cost and sustainability of DSCs [36]. This motivates us and others to explore the use of Earth abundant and cheap metals such as copper [37,38,39,40] and iron [41,42]. In 1998, the first DCSs based on a tris(2,2′-bipyridine)iron(II) complex were reported with a short-circuit current density (*J*_SC_) of 290 μA cm^−2^ [43]. The use of iron(II) complexes for DSC applications is challenging due to their fast deactivation from a metal-to-ligand charge transfer (MLCT) to metal-centred (MC) state [41], which results in inefficient electron injection and low *J*_SC_ values. In 2013, Wärnmark and co-workers [44] published the first iron(II) *N*-heterocyclic carbene (NHC) complex, 1, (Figure 1b) with an extended ^3^MLCT lifetime of 9 ps. Following from this, Gros and co-workers [45] fabricated the first series of iron-sensitized DSCs with the best efficiency (0.13%) known at that time. Currently, the PCEs of DSCs sensitized with copper(I) or iron(II) dyes are considerably lower than those with ruthenium(II) or metal-free dyes. Nevertheless, the benefits of using Earth abundant metals provides an impetus to optimizing their performances to facilitate the development of sustainable materials chemistry.

**Figure 2:**
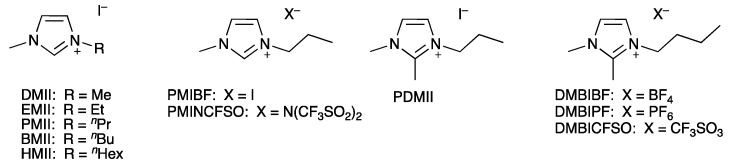
Structures of ILs used in this investigation.

Complex 2 (Figure 1b) is currently the most promising NHC iron-based dye [42,46]. This is despite the fact that 2 is a homoleptic NHC complex, which most likely cannot provide efficient electron injection into the semiconductor because of its fundamental electronic structure and excited state properties [46]. However, tuning of electrolyte composition can also remarkably enhance the PCE and, as shown for other dyes [31], has the potential to make iron-sensitizers a promising alternative to ruthenium-based compounds. It has been demonstrated that both the redox couple and the components of the electrolyte have a critical influence on the PCE, and this effect originates from its role as a charge transfer medium [32,47]. In this investigation, we focus on the electrolyte composition with an I^−^/I_3_^−^ redox shuttle. A conventional liquid electrolyte consists of a redox couple, a solvent, and additives. The term ‘additives’ is used to encompass species such as ionic liquids (ILs), lithium salts (LiX), and various Lewis bases. The redox couple is one of the key constituents of a DSC and it ensures effective dye regeneration. The oxidized form of the redox couple must subsequently diffuse to the counter electrode for reduction [48]. The solvent must allow a fast diffusion of both components of the redox couple, has to solubilize charged species, and should have a low vapour pressure for the long-term stability of cells. It has been shown by Han et al. [49] that the donor abilities of solvents scale with good performances of DSCs by enhancing the open circuit voltage (*V*_OC_). Additives are mainly used to tune the semiconductor conduction band energy [50] for suppressing the rate of recombination of injected electrons from the semiconductor with the electrolyte. The most common additives used in electrolytes are based on guanidine or nitrogen-containing heterocycles, which can move the CB of a semiconductor towards negative potentials. This leads to a significant increase in *V*_OC_ [50,51,52]. The presence of Li^+^ ions also influences the CB due to their adsorption on the surface. However, this effect moves the CB toward more positive potentials, leading to efficient electron injection into the semiconductor but also resulting in a decrease in *V*_OC_ [53]. It is generally recognized that addition of Li^+^ ions improves the photocurrent with ruthenium dyes [31], but, at the same time, it has been shown that, for copper(I)-based DSCs, the presence of LiI is not beneficial [54]. Other common additives to electrolytes are ionic liquids, which could, potentially, substitute for the organic solvent. Advantages of ILs are their thermal stability, high boiling point, and ionic conductivity, which contribute to long DSC lifetimes. On the other hand, high viscosities or the fact that some ILs are solid at 298 K, are disadvantages. IL-based and solvent-free electrolytes have been thoroughly studied by many research groups. The work of Grätzel and co-workers [55] demonstrates that high performing solar cells can be achieved with pure IL electrolytes. In 2008, they reported [56] promising device lifetimes indicating the future potential of ILs. The current challenge of using ILs as electrolyte media is their high viscosity, which results in less effective mass transport. The addition of a co-solvent helps to overcome these limitations and allows the use of ILs with high melting points. The most commonly employed ILs in electrolytes are imidazolium salts, such as those shown in Figure 2. ILs with an iodide counterion in combination with iodine lead to polyiodide structures. In 2015, it was reported that C–H···I–I–I and π···I–I-interactions between these polyiodide anions and imidazolium cations cause a weakening of the I–I bonds resulting in a higher conductivity of ILs [57]. This effect can greatly contribute to the performance of the I^−^/I_3_^−^ redox shuttle and result in more efficient performance of a DSC.

We have been focusing our attention on the effects of using different additives in electrolytes combined with NHC iron-based dyes in DSCs [58]. Recently, we reported that the PCE of DSCs sensitized by compound **2** could be increased from the 0.13% reported by Gros [46] to values in the range of 0.47% to 0.57% by using an I^−^/I_3_^−^-based electrolyte with 3-methoxypropanenitrile (MPN) as solvent and with the IL 1,2-dimethyl-3-propylimidazolium iodide as the only additive [58]. The enhancement in performance was largely associated with significantly increased values of *J*_SC_ ranging from 2.31 to 2.78 mA cm^−2^ [58]. These preliminary investigations motivated us to undertake more detailed investigations of the effects of changing the electrolyte composition while retaining an I^−^/I_3_^−^ redox shuttle and dye 2. Herein, we report electrolyte systems for iron(II) NHC sensitized DSCs, which lead to improved performance and we present the effects of varying the lithium salts and ionic liquid additives. Optimization of electrolyte compositions for metal-free (organic) and ruthenium(II) dyes [16,31,32] and bis(diimine)copper(I) dyes [22,40,54,59] has been a critical part of improving the PCEs of DSCs containing these different types of dyes.

## 2. Materials and Methods

All experimental data including DSC fabrication and electrolyte preparation are given in the Supporting Information. All DSCs contain 3-methoxypropanenitrile as the solvent in electrolytes. Sets of multiple DSCs (all fully masked) were made for each different electrolyte and all data are presented in the Supporting Information. Data presented in tables in the manuscript are representative of the complete data sets and the trends described are consistent for all the multiple sets. The equivalent circuit model used for fitting electrochemical impedance spectroscopy (EIS) experiments is given in Supporting Information (Figure S1).

## 3. Results and Discussion

### 3.1. Influence of Lithium Salts on the Performance of the Fe(II)-NHC DSCs

As discussed above, lithium ions, typically introduced as the salt LiI, are commonly used as an additive in electrolytes in DSCs because they enhance electron injection into the semiconductor [31,53]. The I^−^ counterion may also modify the potential of the I^−^/I_3_^−^ redox shuttle. When a current is flowing, the iodide and triiodide concentrations deviate from equilibrium, resulting in a potential shift, according to the Nernst equation [60]. We, therefore, decided to investigate the effects of incorporating LiI or LiPF_6_ additives and have screened a number of electrolytes, the compositions of which are given in Table 1.

**Figure 3:**
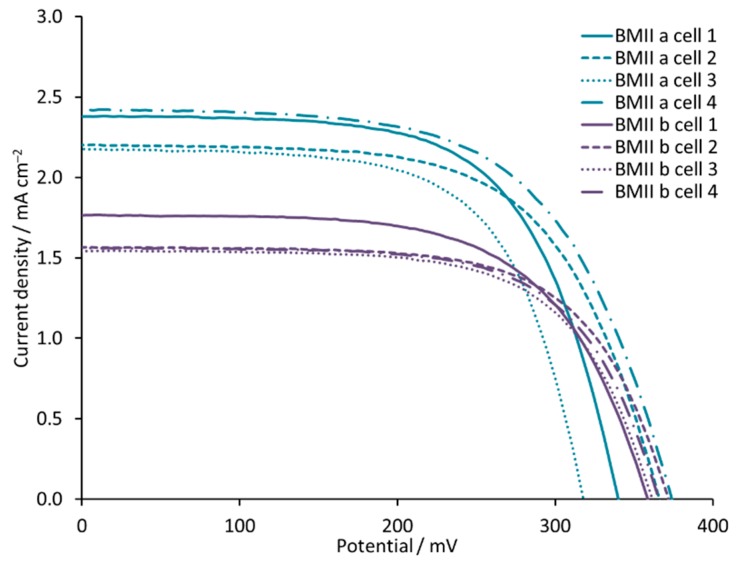
*J-V* curves for sets of multiple DSCs with electrolytes BMIIa and BMIIb.

**Figure 4:**
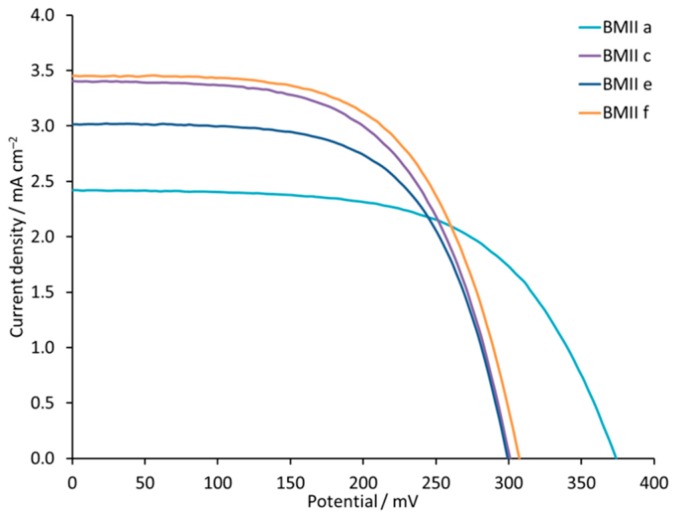
*J-V* curves for the DSCs with electrolytes BMIIa, BMIIc, BMIIe, and BMIIf. Data for multiple DSCs are shown in Figure S2 (see Supporting Information).

Our starting point was an electrolyte composition consisting of LiI (0.1 M), I_2_ (0.05 M), and one of the ionic liquids (0.6 M) 1-propyl-3-methylimidazolium iodide (PMII) or 1-butyl-3-methylimidazolium iodide (BMII). Electrolytes PMIIa and PMIIb, as well as electrolytes BMIIa and BMIIb, differ only in the Li^+^ salt (LiI or LiPF_6_, respectively, Table 1). Interestingly, the trend in the *J*_SC_ values is different for PMII and BMII ILs (Table 2 and Table S2). In the case of PMII, we observe an increase in *J*_SC_ from 2.34 to 2.71 mA cm^−2^ on going from PMIIa to PMIIb, but, for BMII, the *J*_SC_ values decrease from 2.42 mA cm^−2^ for BMIIa to 1.77 mA cm^−2^ for BMIIb. This trend is confirmed with multiple cells (Figure 3). For both ILs, the change from LiI to LiPF_6_ results in lower *V*_OC_, *ff*, and overall PCE. An increase in the concentrations of LiI and LiPF_6_ changes the performance for DSCs with both PMII and BMII ILs. A 0.18 M concentration of Li^+^ ions leads to an increase in *J*_SC_ and PCE compared to a 0.1 M concentration (Figure 4). Electrolytes PMIIc and PMIId both contain 0.18 M Li^+^ but differ in the counterion (Table 1). An enhanced *V*_OC_ of 315 mV and a lower *J*_SC_ of 3.01 mA cm^−2^ for DSCs with PMIIc compared to 281 mV of *V*_OC_ and 3.91 mA cm^−2^ of *J*_SC_ for PMIId result in similar PCE values of 0.59% (10.5% relative to 100% set for N719) for DSCs containing both these electrolytes. We use a relative efficiency because this allows comparisons of data, for example when recorded on different solar simulators or within different laboratories [61]. However, a different trend is observed for BMIIc and BMIId, both of which contain 0.18 M Li^+^ but a different counterion (Table 2). DSCs with BMIIc perform slightly better with PCE of 0.61%, than those with BMIId with PCE of 0.58%. A further increase of LiI concentration from 0.18 M to 0.26 M for DSCs with the BMII based electrolyte is followed by a loss in *J*_SC_ from 3.40 to 3.02 mA cm^−2^ but no change in *V*_OC_ (Table 2, Figure 4, and Figure S2 in Supporting Information). This leads to a value of PCE = 0.56% for the DSC with a BMIIe electrolyte. The change to 0.34 M LiI for BMIIf results in an increase in PCE to 0.64% with *J*_SC_ of 3.45 mA cm^−2^ and *V*_OC_ of 307 mV. The trends in the *J–V* curves for DSCs with electrolytes BMIIc-f are significant because, typically, it is observed that an improvement in either *J*_SC_ or *V*_OC_ is offset by a decrease in *V*_OC_ or *J*_SC_, respectively.

**Figure 5:**
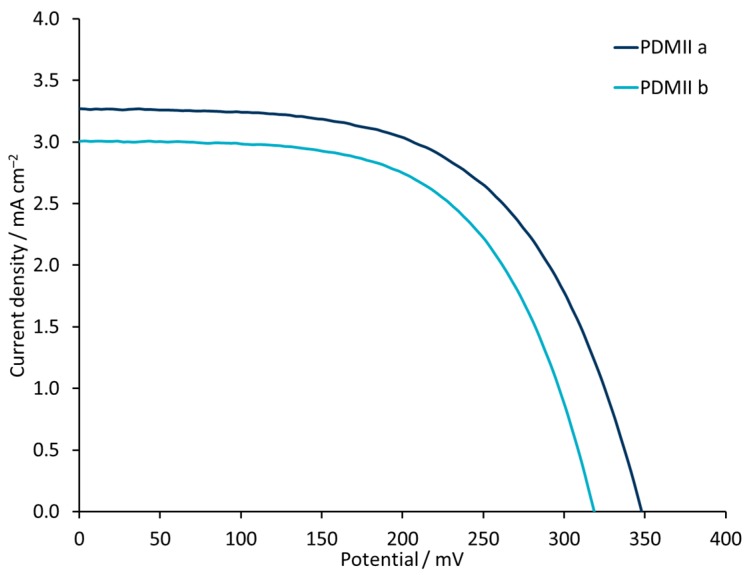
*J-V* curves for the DSCs with electrolytes PDMIIa and PDMIIb. Data for multiple DSCs are shown in Figure S3 (see Supporting Information).

It is important to note that, in the case of ILs with an iodide counterion, both this and LiI contribute I^−^ to the redox couple. In an investigation that focuses on the influence of Li^+^ ions, it is critical that the concentration of I^−^ is constant. The initial electrolyte BMIIa contains LiI (0.1 M) and BMII (0.6 M) with a total iodide concentration of 0.7 M. With an increase of LiI in the electrolyte, the total I^−^ concentration increases as well. To keep it constant, we decreased the amount of IL present in the electrolyte. On going from electrolyte BMIIa (LiI 0.1 M, BMII 0.6 M) to BMIIg (LiI 0.18 M, BMII 0.52 M), BMIIh (LiI 0.26 M, BMII 0.44 M) and BMIIi (LiI 0.34 M, BMII 0.36 M), *J*_SC_ values are enhanced in the range of 2.42–3.61 mA cm^−2^. This rise leads to a better PCE up to 0.63%, despite the loss in *V*_OC_ from 374 to 301 mV for BMIIh. A further increase in LiI and decrease in BMII IL concentration leads to the loss of PCE due to the low *V*_OC_ values of 264 mV. A similar trend is observed for PMIIa and PMIIe electrolytes. The improved *J*_SC_ of 2.80 mA cm^−2^ for PMIIe compared to 2.34 mA cm^−2^ for PMIIa leads to a higher PCE of 0.64%. For the electrolytes with 1-propyl-2,3-dimethylimidazolium iodide IL, a higher concentration of LiI is beneficial. The change from PDMIIa to PDMIIb results in a small decrease of *J*_SC_ from 3.27 to 3.21 mA cm^−2^, of *V*_OC_ from 348 to 337 mV and of PCE from 0.66% to 0.62%, respectively (Figure 5 and Figure S3 in Supporting Information). It is significant to note that a PCE of 0.66% corresponds to a relative efficiency of 11.8% with respect to N719 set at 100% [61], which is the highest yet observed value for an iron(II)-NHC dye.

**Figure 6:**
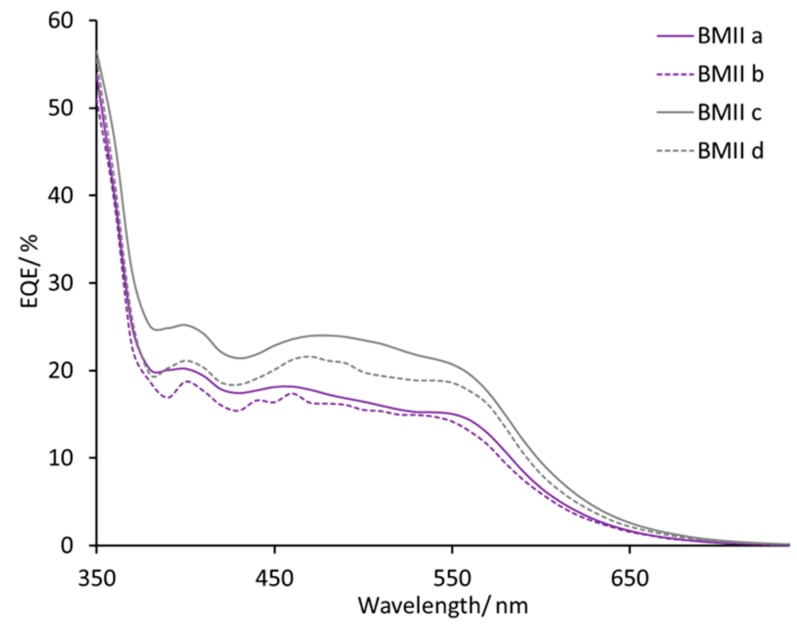
EQE spectra for the DSCs with electrolytes BMIIa, BMIIb, BMIIc, and BMIId. Data for multiple DSCs are shown in Figure S5 (see Supporting Information).

**Figure 7:**
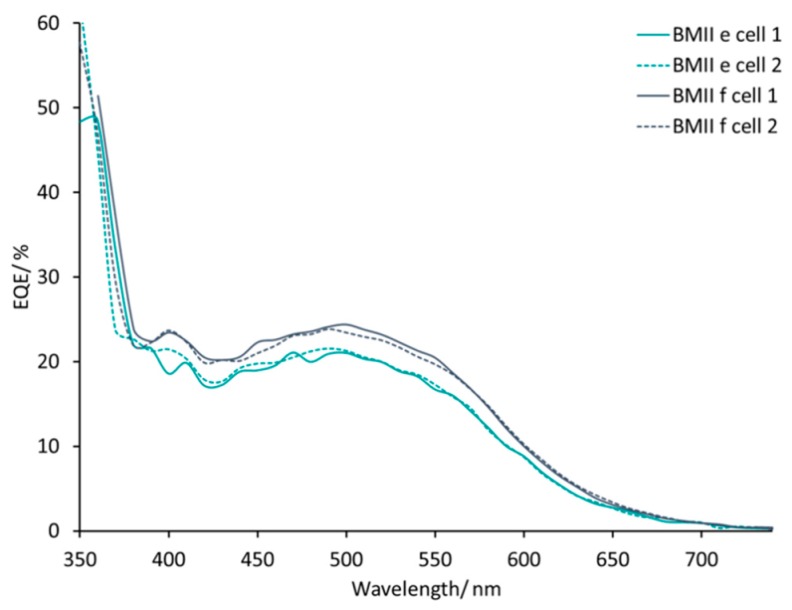
EQE spectra for the multiple DSCs with electrolytes BMIIe and BMIIf.

**Figure 8:**
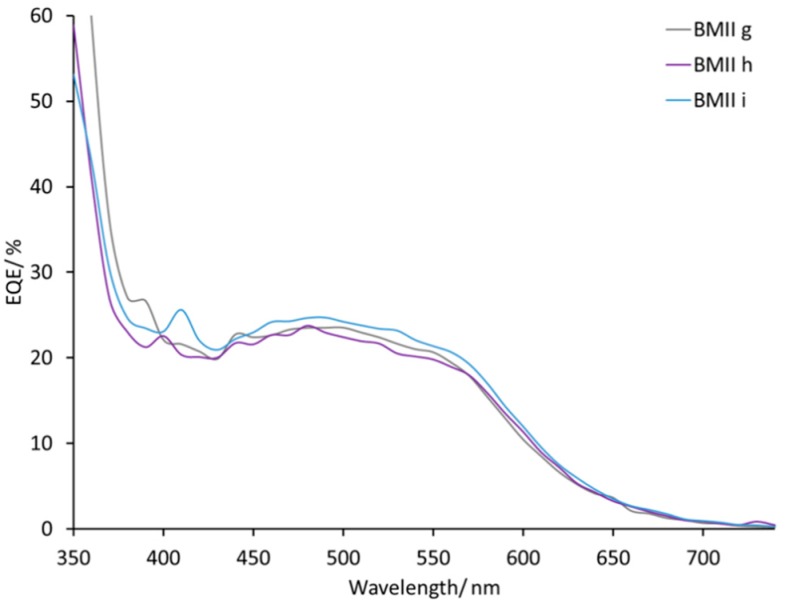
EQE spectra for the DSCs with electrolytes BMIIg, BMIIh, and BMIIi. Data for multiple DSCs are shown in Figure S6 (see Supporting Information).

DSCs with electrolytes from PMIIa to BMIId listed in Table 2 all exhibit broad external quantum efficiency (EQE) spectra in the range of 430–570 nm (Figures S4 and S5) consistent with previous reports [45,54]. The value of EQE_max_ does not change on going from PMIIa to PMIIb and is ≈17–20% at λ_max_ 470–500 nm for a set of four DSCs for each electrolyte. DSCs with electrolytes BMIIa and BMIIb have similar EQE spectra with values of EQE_max_ in the range 12–18% (Figure 6). With an increased concentration of Li^+^ salts, the EQE_max_ increased to 21–23% at λ_max_ = 470 nm for PMII c and up to 20%–27% at λ_max_ = 550 nm for PMIId. For BMII-based electrolytes, higher Li^+^ concentrations also result in higher EQE_max_ values of approximately 24% for BMIIc and of approximately 20% for BMIId at λ_max_ = 470 nm. The increase of LiI concentration from 0.26 to 0.34 M in the electrolytes positively affects the EQE_max_. The EQE_max_ for BMIIe is about 22% and for BMIIf is about 24–25% at λ_max_ 490–510 nm (Figure 7). The EQE spectra of electrolytes BMIIg, BMIIh, and BMIIi can illustrate the influence of Li^+^ ions from 0.18 M to 0.34 M due to the constant concentration of I^−^. On going from BMIIg to BMIIi, the EQE_max_ increases from about 23% to about 25% at λ_max_ 480–500 nm (Figure 8 and Figure S6).

**Figure 9:**
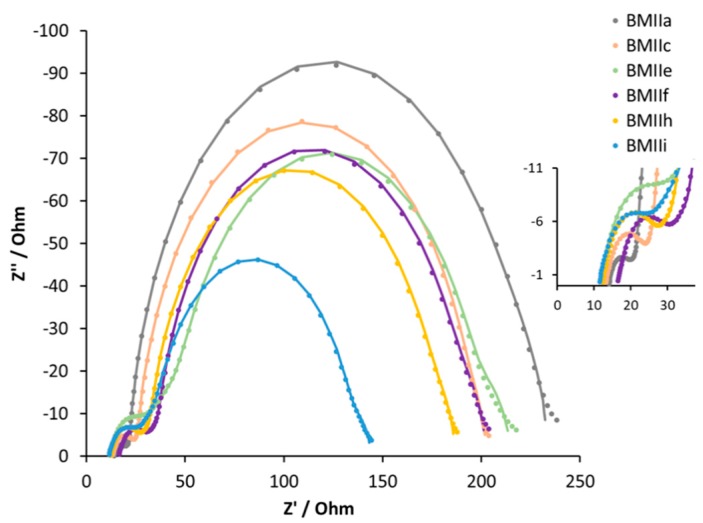
Nyquist plots of the DSCs with electrolytes BMIIa, BMIIc, BMIIe, BMIIf, BMIIh, and BMIIi. Data for multiple DSC sets are shown in Figures S7–S12. Solid lines represent fitted curves. Dotted lines represent experimental data. The expansion shows the high frequency region.

For a deeper understanding of the influence of LiI on the Fe-NHC-sensitized DSC systems, EIS measurements were performed (Table 3, Figure 9 and Figure S12). The series resistance (*R*_s_), and the resistance (*R*_Pt_) and capacitance (*C*_Pt_) of the counter electrodes stay constant for all the DSCs. The recombination resistance (*R*_rec_) and the chemical capacitance (*C*_μ_) decrease with an increase of the Li^+^ ion concentration in the electrolyte up to 0.26 M. For 0.34 M LiI in electrolyte BMIIf, the values of *R*_rec_ and *C*_μ_ increase again. The transport resistance (*R*_tr_) has a corresponding trend with an increase from 9 to 69 Ω from 0.1 M LiI to 0.26 M LiI, but then a decrease in *R*_tr_ to 26 Ω for 0.34 M LiI.

The diffusion length (*L*d), transport time (*τ*_t_), and electron lifetime (*τ*) play an important role in the DSC system. For the effective electron collection throughout the semiconductor, *L*d has to be around three times longer than the thickness of TiO_2_ [62], while *τ*_t_ has to be lower than *τ*. According to reported EIS measurements, high Li^+^ concentrations in the electrolyte can significantly increase *L*d in the case of DSCs with the ruthenium(II)-based Z907 dye [53]. However, for the iron(II)-NHC dye **2**, we observed the opposite trend. In the series 0.1 M to 0.18 M to 0.26 M LiI in the presence of 0.6 M of BMII (electrolytes BMIIa, BMIIc, BMIIe, respectively, see Table 1 and Figures S7–S9), *L*d values decrease from 56 to 18 μm. At the same time, with the reduction of *L*d, *τ* decreases from 74 to 39 ms, while *τ*_t_ increases from 3 to 18 ms for electrolytes BMIIa, BMIIc, and BMIIe. The Bode plot shows the same trend in *τ* values, since the charge lifetime is inversely correlated to the maximum frequency *f*_max_ (Figure S13) [63]. Electrolyte BMIIf is an exception from this trend (Table 3, Figure S10) with the increase in LiI concentration from 0.26 to 0.34 M leading to higher values of *L*d and *τ*, and to a reduction of *τ*_t_.

In the case of DSCs containing electrolytes, BMIIh and BMIIi with a constant 0.7 M iodide ion concentration, the EIS data are consistent with *J*_SC_ and *V*_OC_ trends. Comparing DSCs with electrolytes BMIIe and BMIIh (see Table 1), the decrease in the concentration of the IL from 0.60 to 0.44 M leads to a higher *J*_SC_, which is manifested in lower *R*_rec_ and *R*_tr_. The further increase in the concentration of LiI to 0.34 M and a corresponding decrease of the BMII concentration to 0.36 M (to maintain a constant [I^−^]) in the BMIIi electrolyte leads to lower *R*_rec_ and *C*_μ_ values, but higher *R*_tr_ compared to values observed when the electrolyte is BMIIh (Table 3).

The above results demonstrate that an increase in the concentration of Li^+^ ions positively affects the PCE for all ILs. The *J–V* and EIS data for DSCs containing electrolytes BMIIc and BMIIf are very similar with a slightly higher PCE when BMIIf is used. Based on these results, we were motivated to extend the investigations using 0.18 M LiI as the optimal additive.

### 3.2. Influence of the Structure of the Ionic Liquid

The structure of the IL in the electrolyte can be crucial in terms of DSC performance. The change in an alkyl chain length of 1-alkyl-3-methylimidazolium iodide family results in different viscosity, conductivity, and diffusion properties [64]. We chose five ILs based on methylimidazolium iodide with different alkyl chain lengths 1,3-dimethylimidazolium iodide, 1-ethyl-3-methylimidazolium iodide, 1-propyl-3-methylimidazolium iodide, 1-butyl-3-methylimidazolium iodide, and 1-hexyl-3-methylimidazolium iodide (Figure 2).

**Figure 10:**
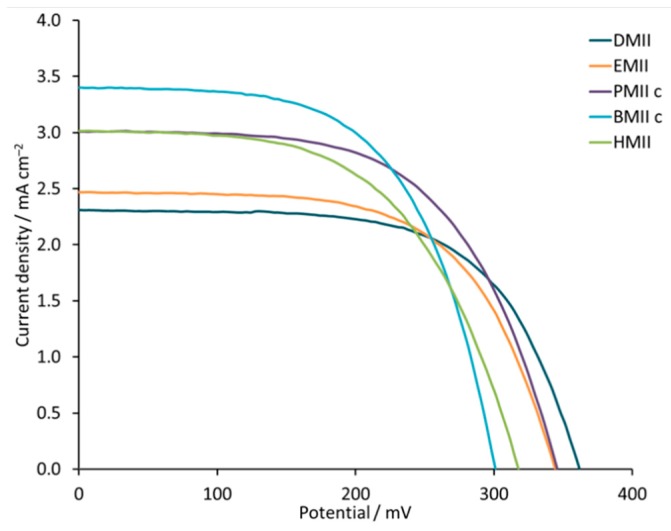
*J-V* curves for the DSCs with electrolytes DMII, EMII, PMIIc, BMIIc, and HMII. Data for multiple DSCs are shown in Figure S14 (see Supplementary Information).

Table 4 and Table S2 (see Supporting Information) give the measured parameters for DSCs containing electrolytes incorporating the different ILs. In each case, the electrolyte composition was LiI (0.18 M), I_2_ (0.05 M), and IL (0.6 M) in MPN. We observed an increase in PCE as the alkyl chain lengthened. From DMII to BMIIc, an increase in *J*_SC_ from 2.31 to 3.40 mA cm^−2^ is observed, but this is countered by a loss in *V*_OC_ from 362 to 301 mV (Table 4 and Figure 10). The higher *J*_SC_ values result in a PCE of 0.61% (which represents a noteworthy value of 10.9% relative to 100% set for N719 [61]) for BMIIc despite the reduction in *V*_OC_ compared to DMII. DSCs with electrolyte HMII appear out of line with the trend observed for the shorter alkyl chains (Table 4 and Figure 9). A decrease in *J*_SC_ to 3.14 mA cm^−2^ is compensated by a slightly higher *V*_OC_ of 316 mV and leads to a similar overall performance (PCE = 0.60%) as for BMIIc.

**Figure 11:**
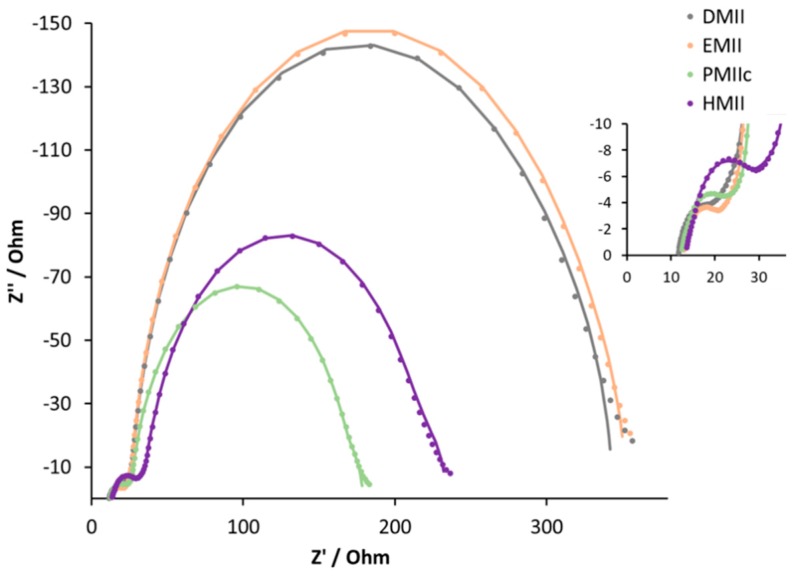
Nyquist plots of the DSCs with electrolytes DMII, EMII, PMIIc, and HMII. Data for multiple DSC sets are shown in Figures S15–S18 (see Supporting Information). Solid lines represent fitted curves and dotted lines represent experimental data. The expansion shows the high frequency region.

The results of EIS experiments (Table 5, Figure 11, and Figures S15–S18) are consistent with the observed trends in DSC performances. On going from the methyl to n-propyl substituent, we observe a decrease in both the recombination resistance and the chemical capacitance. On going from the electrolyte with DMII to EMII, there is a slightly higher Cμ combined with a smaller transport resistance. This explains the small difference in VOC and JSC values for these DSC sets and results in a similar overall performance. The Cμ values for PMIIc and BMIIc are comparable, but higher Rrec in combination with smaller Rtr for BMIIc lead to a more favourable electron injection in the semiconductor. This effect results in a higher JSC value and, as a consequence, in a better DSC efficiency in the case of the BMII electrolyte. The change from n-butyl to n-hexyl chain does not lead to any significant difference in Rrec and Cμ, and, therefore, results in similar PCE values. The values of Ld, τt, and τ, in the case of the DSCs in Table 4, are consistent with each other. The trend in τ is confirmed with the Bode plot (Figure S19). Low values of τt and Rtr for DSCs with the BMII electrolyte represent a small electron loss in the TiO2 semiconductor. Together, these parameters result in well-performing DSCs.

**Figure 12:**
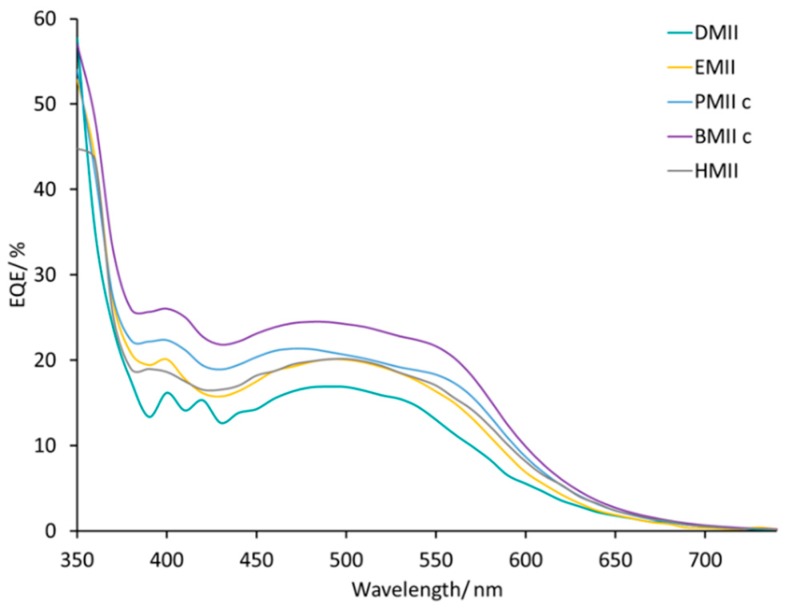
EQE spectra for the DSCs with electrolytes DMII, EMII, PMIIc, BMIIc, and HMII. Data for multiple DSCs are shown in Figure S20 (see Supporting Information).

The EQE spectra of the DSCs listed in Table 4 cover a broad range of wavelengths (Figure S20). The change from methyl to *n-*butyl chain enhances the EQE_max_ up to 24% (λ_max_ = 430 to 570 nm) and this is consistent with the trend in *J*_SC_. The use of the HMII-based electrolyte leads to the same EQE_max_ value (≈20%) as observed for DSCs with EMII (Figure 12). Thus, we conclude that, of the imidazolium iodides screened, incorporation of an *n*-butyl side-chain contributes to the best efficiency DSCs with optimal values of *V*_OC_, *J*_SC_, EQE_max_, and EIS parameters.

### 3.3. Influence of Ionic Liquid Counterions

**Figure 13:**
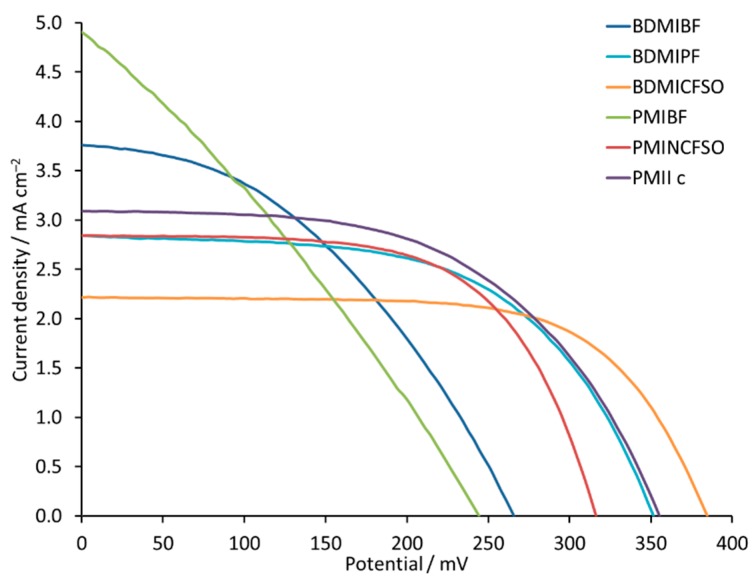
*J-V* curves for DSCs with electrolytes BDMIBF, BDMIPF, BDMICFSO, PMIBF, PMINCFSO, and PMIIc (see Figure 2 for abbreviations). Data for multiple DSCs are shown in Figure S21 in the Supporting Information.

The iodide counterion contributes to a faster dye regeneration due to the increased concentration of I^−^ in the system [65]. On the other hand, too high a concentration of imidazolium iodide leads to a loss of the potential photocurrent [65]. This drawback motivated us to investigate the effect of different counterions in the ILs in the electrolytes. We have focused on 1-butyl-2,3-dimethylimidazolium salts with tetrafluoridoborate, hexafluoridophosphate, and trifluoromethanesulfonate counterions and 1-propyl-3-methylimidazolium salts with tetrafluoridoborate, bis(trifluoromethylsulfonyl)imide, and iodide counterions (Figure 2). The performances of DSCs containing the different electrolytes are summarized in Figure 13 and Table 6, and in Table S5 and Figure S21 in the Supporting Information. In the sequence of [BDMI]^+^ salts, values of *J*_SC_ fall from 3.80 to 2.22 mA cm^−2^, while *V*_OC_ increases from 266 to 385 mV. In the [PMI]^+^ series, the same trend is observed. For PMIBF, PMINCFSO, and PMIIc, the decrease in *J*_SC_ values is compensated by an increase in *V*_OC_, which results in a PCE for the DSCs in the range of 0.35% to 0.59%. A PCE of 0.59% translates to a relative efficiency of 10.5% with respect to N719 set at 100%, which is rather high for an iron-based sensitizer. The change from [PF_6_]^−^ to [CF_3_SO_3_]^−^ for [BDMI]^+^ has little effect on the overall DSC performances (0.57% and 0.56%), while on going from [N(CF_3_SO_2_)_2_]^−^ to I^−^ for [PMI]^+^ (i.e., PMINCFSO to PMIIc, Table 6) there is a moderate gain in performance from 10.0% to 10.5% relative to N719 [61], as a consequence of values of *J*_SC_ = 3.01 mA cm^−2^ and *V*_OC_ = 315 mA. In the case of the [BF_4_]^−^ counterion for both BDMIBF and PMIBF, the low *ff* values of 41% and 30% in combination with low *V*_OC_ values of 266 and 264 mV result in PCE values of 0.41% and 0.35%, respectively (Table 6). This suggests that use of [BF_4_]^−^ containing ILs is not beneficial. When the counterion is changed to [PF_6_]^−^, the *ff* increases and the overall performance of the cells improves.

## 4. Conclusions

In this study, we report the enhanced performances of DSCs containing the homoleptic iron(II) sensitizer **2** and I^−^/I_3_^−^ redox shuttle by variation in the composition of the electrolyte. An increased concentration of Li^+^ positively affects the overall DSC performance and the EQE_max_. Values of *J*_SC_ = 3.61 mA cm^−2^ and EQE_max_ ≈ 25% are observed for electrolyte BMIIi, which contains 0.34 M LiI and 0.36 M BMII in 3-methoxypropanenitrile. The combination of 0.60 M PDMII IL and 0.18 M LiI lead to a PCE up to 0.66%, which is 11.8% relative to 100% set for N719. Currently, this is the highest observed overall efficiency for an iron(II)-NHC sensitizer. All DSCs in this investigation were fully masked to prevent overestimations of PCEs.

An investigation of the effects of varying the alkyl chain length of 1-alkyl-3-methylimidazolium iodide ILs has shown that longer alkyl chains result in an increase in *J*_SC_ from 2.31 to 3.40 mA cm^−2^ and PCE from 0.53% to 0.61% on going from *n*-methyl to *n*-butyl chains. A further lengthening to an *n*-hexyl chain results in a similar PCE value of 0.60% (10.7% relative to N719 set at 100%) with slightly lower *J*_SC_ of 3.14 mA cm^−2^ compared to 3.40 mA cm^−2^ for BMII IL. The EQE_max_ also increases in the order DMII > EMII > PMII > BMII from ≈17% to ≈24%. The use of HMII IL reverses the trend with a lower EQE_max_ of ≈20%. R_tr_ values extracted from EIS measurements confirm the trend in *J*_SC_ and EQE_max_.

A change of the counterion in the IL from I^−^ did not lead to any increase in performance of the DSC. Use of the [BF_4_]^−^ counterion leads to DSCs with the poorest performances. Despite a high *J*_SC_ of 3.80 and 4.90 mA cm^−2^ for BDMIBF and PMIBF, *ff* values of 41 and 30% in combination with *V*_OC_ values of 266 and 244 mV result in low PCE values of 0.41 and 0.35%, respectively. Use of other counterions [PF_6_]^−^ and [CF_3_SO_3_]^−^ for [BDMI]^+^ IL and [N(CF_3_SO_3_)_2_]^−^ for [PMI]^+^ leads to DSCs that perform similarly with PCEs of 0.56–0.57% (10.0–10.2% relative to 100% for N719).

In conclusion, we have shown that tuning of electrolyte additives has a significant impact on iron(II)-sensitized DSCs, leading to performances that approach those of cells sensitized with copper(I)-based dyes [37]. The overall performances as well as the EQE spectra significantly increase with an increase in the concentration of Li^+^ ions in combination with an optimized length of the alkyl-side chain in 1-alkyl-3-methylimidazolium iodide ILs and use of iodide as the counterion. For further improvement of the performances of iron(II)-based DSCs, it is imperative that dye, a redox shuttle, and electrolyte components are optimized.

## Figures and Tables

**Table 1:** Electrolyte compositions with different Li^+^ salts and IL concentrations. The solvent is MPN.

Electrolyte ^1^	LiI/M	LiPF_6_/M	I_2_/M	IL/M
PMIIa	0.10	–	0.05	PMII/0.60
PMIIb	–	0.10	0.05	PMII/0.60
PMIIc	0.18	–	0.05	PMII/0.60
PMIId	–	0.18	0.05	PMII/0.60
BMIIa	0.10	–	0.05	BMII/0.60
BMIIb	–	0.10	0.05	BMII/0.60
BMIIc	0.18	–	0.05	BMII/0.60
BMIId	–	0.18	0.05	BMII/0.60
BMIIe	0.26	–	0.05	BMII/0.60
BMIIf	0.34	–	0.05	BMII/0.60
BMIIg	0.18	–	0.05	BMII/0.52
BMIIh	0.26	–	0.05	BMII/0.44
BMIIi	0.34	–	0.05	BMII/0.36
PDMIIa	0.18	–	0.05	PDMII/0.60
PDMIIb	0.18	–	0.05	PDMII/0.52
PMIIe	0.18	–	0.05	PMII/0.52

^1^ IL abbreviations defined in Figure 2.

**Table 2:** Electrolyte compositions with different Li^+^ salts and IL concentrations. The solvent is MPN.

Electrolyte ^1^	*J*_SC_/mA cm^−2^	*V*_OC_/mV	*ff*/%	η/%	Rel. η/% ^2^
PMIIa	2.34	371	66	0.57	10.2
PMIIb	2.71	264	57	0.41	7.3
PMIIc	3.01	315	62	0.59	10.5
PMIId	3.91	281	53	0.59	10.5
BMIIa	2.42	374	61	0.55	9.8
BMIIb	1.77	359	62	0.39	6.9
BMIIc	3.40	301	59	0.61	10.9
BMIId	3.13	315	59	0.58	10.4
BMIIe	3.02	299	62	0.56	10.0
BMIIf	3.45	307	60	0.64	11.4
BMIIg	3.33	298	62	0.62	11.1
BMIIh	3.46	301	60	0.63	11.3
BMIIi	3.61	264	62	0.59	10.5
PDMIIa	3.27	348	58	0.66	11.8
PDMIIb	3.21	337	57	0.62	11.1
PMIIe	2.80	368	62	0.64	11.4
Reference cell with dye N719	12.53	654	68	5.60	100

^1^ The electrolyte compositions are given in Table 1. ^2^ All DSCs are referenced to an N719 sensitized DSC and relative efficiencies are given with *η* for N719 set to 100% [61].

**Table 3:** EIS parameters for DSCs using electrolytes with different concentrations of LiI. Data for multiple DSCs are given in Table S2 (see Supporting Information).

Electrolyte ^1^	*R*_rec_/Ω	*C*_μ_/μF	*R*_tr_/Ω	*τ*/ms	*τ*_t_/ms	*L*d/μm	*R*_s_/Ω	*R*_Pt_/Ω	*C*_Pt_/μF
BMIIa	192	384	9	74	3	56	14	4	6
BMIIc	169	307	13	52	4	43	13	8	6
BMIIe	150	258	69	39	18	18	11	11	7
BMIIf	152	374	26	57	10	29	16	11	5
BMIIh	144	378	23	54	9	30	12	12	5
BMIIi	99	302	40	30	12	19	11	10	6

^1^ The electrolyte compositions are given in Table 1.

**Table 4:** Parameters for DSCs using electrolytes based on imidazolium ionic liquids with different lengths of a side chain. Data for multiple DSCs are given in Table S3 in the Supporting Information section.

Electrolyte ^1,2^	*J*_SC_/mA cm^−2^	*V*_OC_/mV	*ff*/%	*η*/%	Rel. *η*/% ^3^
DMII	2.31	362	63	0.53	9.5
EMII	2.47	344	62	0.52	9.3
PMIIc	3.01	315	62	0.59	10.5
BMIIc	3.40	301	59	0.61	10.9
HMII	3.14	316	60	0.60	10.7
Reference cell with dye N719	12.53	654	68	5.60	100

^1^ The electrolyte compositions are LiI (0.18 M), I_2_ (0.05 M), and IL (0.6 M) in MPN. ^2^ Data for PMIIc and BMIIc are repeated from Table 2 for convenience. ^3^ All DSCs are referenced to N719 sensitized DSC. Relative efficiencies are given with N719 PCE set to 100% [61].

**Table 5:** EIS parameters for DSCs using electrolytes based on imidazolium ionic liquids with different lengths of the side-chain. Data for multiple DSCs are given in Table S4 (see Supporting Information).

Electrolyte ^1,2^	*R*_rec_/Ω	*C*_μ_/μF	*R*_tr_/Ω	*τ*/ms	*τ*_t_/ms	*L*d/μm	*R*_s_/Ω	*R*_Pt_/Ω	*C*_Pt_/μF
DMII	269	416	22	112	9	42	12	6	7
EMII	299	464	19	139	9	48	13	6	5
PMIIc	141	297	19	42	6	33	12	8	5
BMIIc	169	307	13	52	4	43	13	8	6
HMII	179	328	30	59	10	29	13	11	5

^1^ The electrolyte compositions are given in the footnote to Table 4. ^2^ Data for BMIIc are repeated from Table 3 for convenience.

**Table 6:** Parameters for DSCs using electrolytes based on imidazolium ionic liquids with different counterions. Data for multiple DSCs are given in Table S5 in the Supporting Information.

Electrolyte ^1,2^	*J*_SC_/mA cm^−2^	*V*_OC_/mV	*ff*/%	*η*/%	Rel. *η*/% ^3^
BDMIBF	3.80	266	41	0.41	7.3
BDMIPF	2.84	351	58	0.57	10.2
BDMICFSO	2.22	385	66	0.56	10.0
PMIBF	4.90	244	30	0.35	6.3
PMINCFSO	2.84	316	62	0.56	10.0
PMII c	3.01	315	62	0.59	10.5
Reference cell with dye N719	12.53	654	68	5.60	100

^1^ The electrolyte compositions are LiI (0.18 M), I_2_ (0.05 M), and IL (0.6 M) in MPN. ^2^ Data for PMIIc and BMIIc are repeated from Table 2 for convenience. ^3^ All DSCs are referenced to N719 sensitized DSC. Relative efficiencies are given with N719 PCE set to 100% [61].

## Supplementary Materials

The following are available online at https://www.mdpi.com/1996-1944/12/24/4181/s1. Full experimental details. Figure S1: Equivalent circuit model used for fitting EIS data. Figures S2–S3: *J–V* curves for multiple DSCs. Figures S4–S6: EQE spectra for multiple DSCs. Figures S7–S13: EIS data and Bode plots for multiple DSCs. Figure S14: *J–V* curves for multiple DSCs. Figures S15–S19: EIS data and Bode plots for multiple DSCs. Figure S20: EQE spectra for multiple DSCs. Figure S21: *J–V* curves for multiple DSCs. Table S1: Parameters for multiple DSCs using electrolytes with different concentrations of Li^+^ salts and ILs. Table S2: EIS parameters for multiple DSCs using electrolytes with different concentrations of LiI. Table S3: DSC parameters for multiple cells with different ILs. Table S4: EIS parameters for multiple cells with different ILs. Table S5: DSC parameters for multiple cells.
