# Supplementary material for: There Is a Future for N-Heterocyclic Carbene Iron(II) Dyes in Dye-Sensitized Solar Cells: Improving Performance through Changes in the Electrolyte
Source: Materials (Basel). 2019 Dec 12;12(24):4181. doi: 10.3390/ma12244181 (PMC6947502; doi:10.3390/ma12244181)
Supplement: Supplementary file 1 [file materials-12-04181-s001.pdf]

materials

MDPI

Supporting Information

for

There is a Future for *N*-Heterocyclic Carbene Iron(II) Dyes in Dye-Sensitized Solar Cells: Improving Performance Through Changes in the Electrolyte

Mariia Karpacheva, Vanessa Wyss, Catherine E. Housecroft and Edwin C. Constable

Table of Contents

Fabrication of DSCs........ 3

Electrolyte preparation **3**

EIS measurements........ 3

Tables and figures 4

Table S1. Parameters for multiple DSCs using electrolytes with different concentration of lithium salts and ILs. **4**

Fig. S1. The equivalent circuit model used for fitting EIS experiments. **5**

Fig. S2. J-V curves for the multiple DSCs with electrolytes BMIIa, BMIIc, BMIIe and BMIIf. **5**

Fig. S3. J-V curves for the multiple DSCs with electrolytes PDMIIa and PDMIIb. **5**

Fig. S4. EQE spectra for multiple DSCs with electrolytes PMIIa, PMIIb, PMIIc and PMIId **6**

Fig. S5. EQE spectra for multiple DSCs with electrolytes BMIIa, BMIIb, BMIIc and BMIId. **6**

Fig. S6. EQE spectra for multiple DSCs with electrolytes BMIIg, BMIIh and BMIIi. **7**

Fig. S7. EIS data of the multiple DSCs with electrolyte BMIIa. **8**

Fig. S8. EIS data of the multiple DSCs with electrolyte BMIIc. **9**

Fig. S9. EIS data of the multiple DSCs with electrolyte BMIIe. **10**

Fig. S10. EIS data of the multiple DSCs with electrolyte BMIIf. **11**

Fig. S11. EIS data of the multiple DSCs with electrolyte BMIIh. **12**

Fig. S12. EIS data of the multiple DSCs with electrolyte BMIIi. **13**

Fig. S13. Bode plot of the DSCs with electrolytes BMIIa, BMIIc, BMIIe, BMIIf, BMIIh, BMIIi. **14**

Table S2. EIS parameters for multiple DSCs using electrolytes with different concentrations of LiI. **14**

Fig. S14. J-V curves for the multiple DSCs with electrolytes DMII, EMII, PMIIc, BMIIc and HMII. **15**

Table S3. Parameters for multiple DSCs using electrolytes based on imidazolium ionic liquids with different lengths of side-chain. **15**

Fig. S15. EIS data of the multiple DSCs with electrolyte DMII. **16**

Fig. S16. EIS data of the multiple DSCs with electrolyte EMII. 17

Fig. S17. EIS data of the multiple DSCs with electrolyte PMIIc. 18

Fig. S18. EIS data of the multiple DSCs with electrolyte HMII. 19

Fig. S19. Bode plot of the DSCs with electrolytes BMIIa, BMIIc, BMIIe, BMIIf, BMIIh, BMIIi. 20

Table S4. EIS parameters for multiple DSCs using electrolytes based on imidazolium ionic liquids with different lengths of side-chain. 20

Fig. S20. EQE spectra for multiple DSCs using electrolytes DMII, EMII, PMIIc, BMIIc and HMII. 21

Fig. S21. J-V curves for the multiple DSCs with electrolytes based on imidazolium ionic liquids with different counter ions. 21

Table S5. Parameters for multiple DSCs using electrolytes based on imidazolium ionic liquids with different counter ions. 22

Fabrication of DSCs

Each working commercial TiO_2 electrode (opaque, Solaronix) was rinsed with EtOH and dried on a heating plate at 450 °C for 30 min. The electrodes were cooled to 60 °C and immersed in an iron(II) dye **2** (0.5 mM) acetonitrile solution containing chenodeoxycholic acid (0.1 mM) overnight. The reference working electrode was made by dipping a commercial electrode in an 0.3 mM EtOH solution of N719 (Solaronix) overnight. After soaking in the dye-baths, the electrodes were washed with the same solvent as used in the dye-bath (in case of MeCN the electrodes were washed second time with acetone) and dried with a heat gun.

Commercial counter electrodes from Solaronix (Test Cell Platinum Electrodes Drilled) were rinsed with EtOH and dried on a heating plate at 450 °C for 30 min. The TiO_2 electrodes and Pt counter-electrodes were assembled using thermoplast hot-melt sealing foil (Solaronix, Test Cell Gaskets, made from Meltonix 1170-60 sealing film, 60 microns thick) by heating while pressing them together. The electrolytes were introduced into DSCs by vacuum backfilling through a hole drilled in the counter electrode and this was then sealed using hot-melt sealing foil and a cover glass.

The solar cell measurements used fully masked cells using black coloured copper sheet with a single aperture placed over the screen printed dye-sensitized TiO_2 square. The area of the aperture in the mask was smaller than the active area of the dye-sensitized TiO_2 (0.36 cm^2). For complete masking, black tape was also applied over the edges and rear of the cell. Current density-voltage (*J*–*V*) measurements were made by irradiating from behind with a LOT Quantum Design LS0811 instrument (100 mW cm^{-2} = 1 sun at AM 1.5) and the simulated light power was calibrated with a silicon reference cell.

The external quantum efficiency (EQE) measurements were performed on a Spe-Quest quantum efficiency setup from Rera Systems (Netherlands) equipped with a 100W halogen lamp (QTH) and a lambda 300 grating monochromator from Lot Oriel. The monochromatic light was modulated to 1 Hz using a chopper wheel from ThorLabs. The cell response was amplified with a large dynamic range IV converter from CVI Melles Griot and then measured with a SR830 DSP Lock-In amplifier from Stanford Research.

Electrolyte preparation

The electrolyte compositions were as given in Tables 1, 4 and 6. BMII was purchased from Sigma-Aldrich, PMII, EMII, HMII, BDMIBF, BDMIPF, PMIBF, PMINCFSO were purchased from TCI, DMII and PDMII were purchased from Fluorochem, BDMICFSO and MPN were bought from Acros Organics and Fluka, respectively.

EIS measurements

For the EIS measurements a ModuLab® XM PhotoEchem photoelectrochemical measurement system from Solartron Analytical was used. The impedance was measured at the open-circuit potential of the cell at a light intensity of 22 mW cm^{-2} (590 nm) in the frequency range 0.05 Hz to 100 kHz using an amplitude of 10 mV. The impedance data were analysed using ZView® sofware from Scribner Associates Inc.

Tables and figures

Table S1. Parameters for multiple DSCs using electrolytes with different concentration of lithium salts and ILs.

Electrolyte	J_{SC} / mA cm^{-2}	V_{OC} / mV	*ff* / %	*η* / %	Rel. *η* / %
PMIIa cell 1	2.34	371	66	0.57	10.2
PMIIa cell 2	2.26	354	66	0.53	9.5
PMIIa cell 3	2.41	355	65	0.56	10.0
PMIIa cell 4	2.69	373	63	0.63	11.3
PMIIb cell 1	2.71	264	57	0.41	7.3
PMIIb cell 2	2.35	285	60	0.40	7.1
PMIIb cell 3	2.66	265	58	0.41	7.3
PMIIb cell 4	2.77	288	60	0.48	8.6
PMIIc cell 1	3.01	315	62	0.59	10.5
PMIIc cell 2	2.98	270	62	0.50	8.9
PMIIc cell 3	2.98	318	63	0.59	10.5
PMIIc cell 4	-	-	-	-	-
PMIId cell 1	3.91	281	53	0.59	10.5
PMIId cell 2	3.44	296	54	0.55	9.8
PMIId cell 3	3.45	301	56	0.58	10.4
PMIId cell 4	2.85	284	60	0.48	8.6
BMIIa cell 1	2.42	374	61	0.55	9.8
BMIIa cell 2	2.38	340	64	0.52	9.3
BMIIa cell 3	2.20	366	63	0.51	9.1
BMIIa cell 4	2.18	318	64	0.45	8.0
BMIIb cell 1	1.77	359	62	0.39	6.9
BMIIb cell 2	1.54	361	65	0.37	6.6
BMIIb cell 3	1.56	366	66	0.38	6.8
BMIIb cell 4	1.57	372	66	0.38	6.8
BMIIc cell 1	3.40	301	59	0.61	10.9
BMIIc cell 2	3.56	311	57	0.63	11.3
BMIIc cell 3	3.51	291	58	0.59	10.5
BMIIc cell 4	3.40	294	60	0.60	10.7
BMIId cell 1	3.13	315	59	0.58	10.4
BMIId cell 2	2.65	313	62	0.51	9.1
BMIId cell 3	2.92	307	61	0.55	9.8
BMIId cell 4	3.10	330	60	0.61	10.9
BMIIe cell 1	3.02	299	62	0.56	10.0
BMIIe cell 2	2.79	288	64	0.51	9.1
BMIIe cell 3	3.08	286	62	0.55	9.8
BMIIe cell 4	3.10	306	63	0.60	10.7
BMIIf cell 1	3.45	307	60	0.64	11.4
BMIIf cell 2	3.49	302	62	0.65	11.6
BMIIf cell 3	3.38	298	61	0.62	11.1
BMIIf cell 4	3.46	292	61	0.61	10.9
BMIIg cell 1	3.33	298	62	0.62	11.1
BMIIg cell 2	3.03	272	63	0.52	9.3
BMIIg cell 3	3.62	293	60	0.64	11.4
BMIIg cell 4	3.35	277	61	0.57	10.2
BMIIh cell 1	3.46	301	60	0.63	11.3
BMIIh cell 2	3.58	263	47	0.44	7.9
BMIIh cell 3	3.86	311	55	0.66	11.8
BMIIh cell 4	3.32	281	58	0.54	9.6
BMIIi cell 1	3.61	264	62	0.59	10.5
BMIIi cell 2	3.98	264	61	0.64	11.4
BMIIi cell 3	3.49	246	60	0.52	9.3
BMIIi cell 4	3.78	220	60	0.50	8.9
PDMIIa cell 1	3.27	348	58	0.66	11.8
PDMIIa cell 2	3.41	346	54	0.64	11.4
PDMIIb cell 1	3.21	337	57	0.62	11.1
PDMIIb cell 2	3.01	318	60	0.57	10.2
PMIIe cell 1	2.80	368	62	0.64	11.4
PMIIe cell 2	2.71	390	60	0.64	11.4
PMIIe cell 3	2.80	360	62	0.62	11.1
PMIIe cell 4	2.70	366	63	0.62	11.1
N719	12.53	654	68	5.60	100

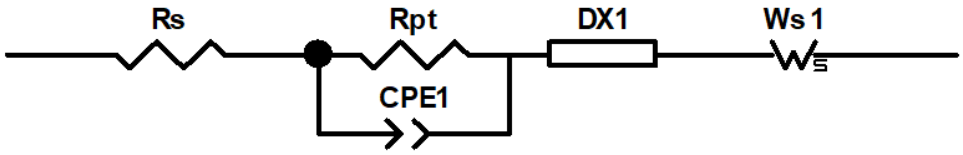

Fig. S1. The equivalent circuit model used for fitting EIS experiments.

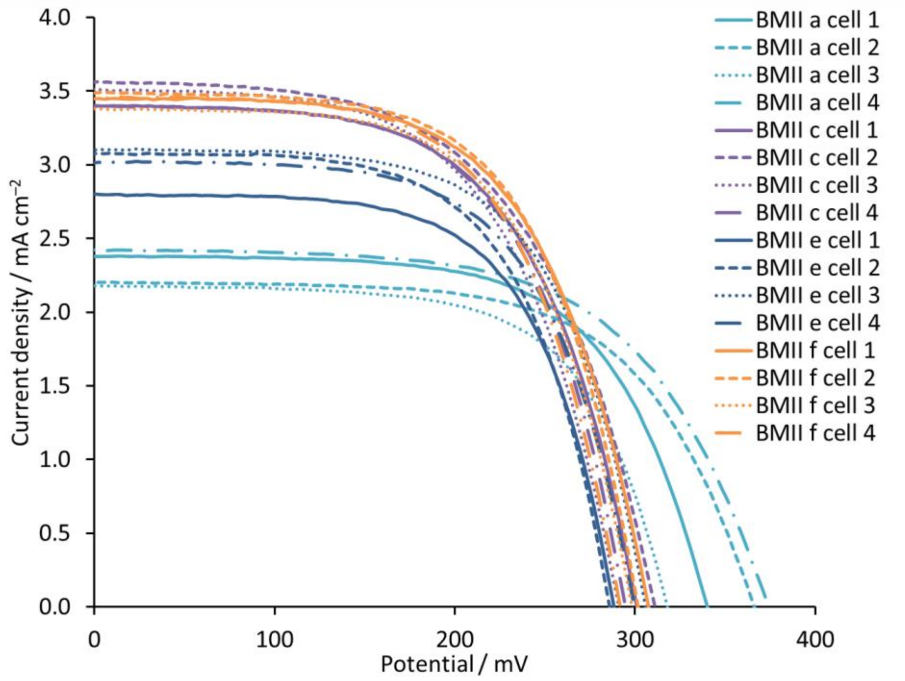

Fig. S2. J-V curves for the multiple DSCs with electrolytes BMIIa, BMIIc, BMIIe and BMIIf.

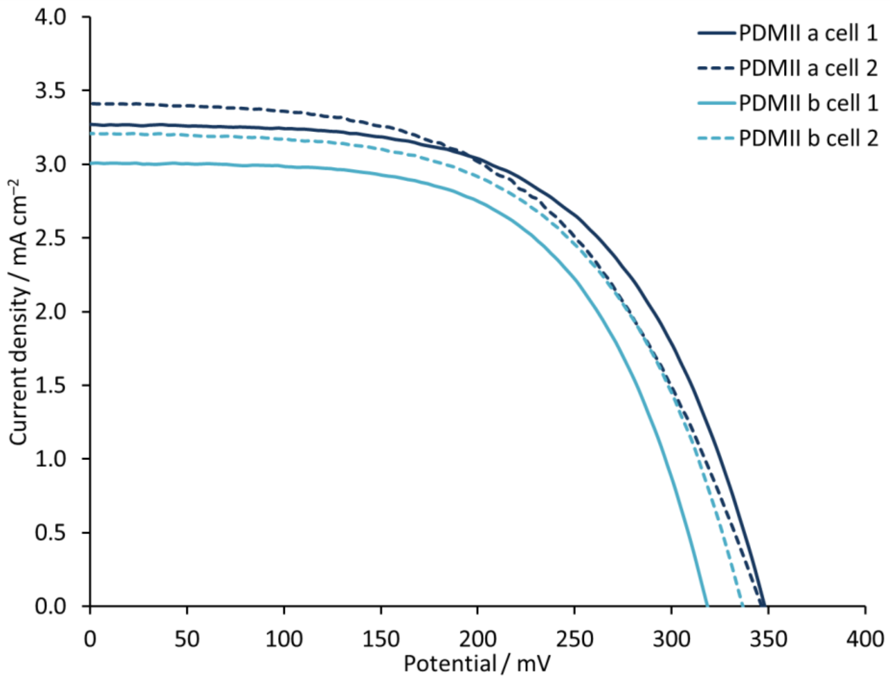

Fig. S3. J-V curves for the multiple DSCs with electrolytes PDMIIa and PDMIIb.

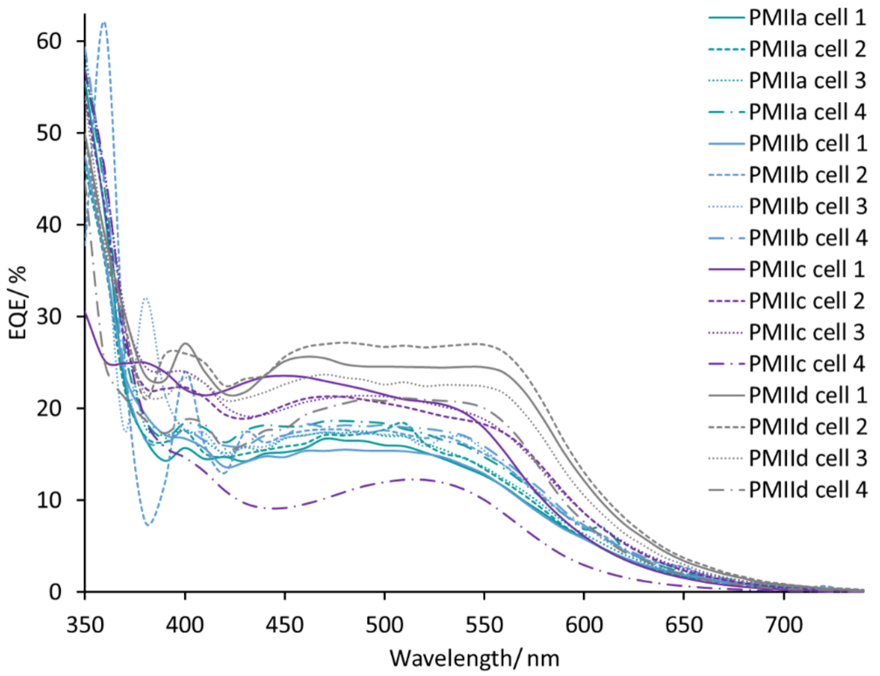

Fig. S4. EQE spectra for multiple DSCs with electrolytes PMIIa, PMIIb, PMIIc and PMIId

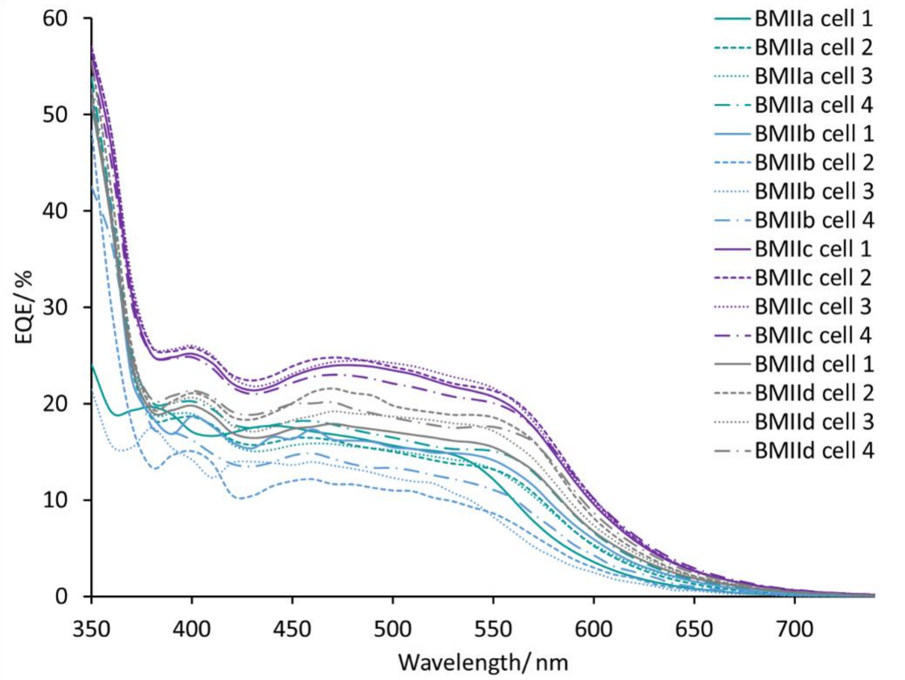

Fig. S5. EQE spectra for multiple DSCs with electrolytes BMIIa, BMIIb, BMIIc and BMIId.

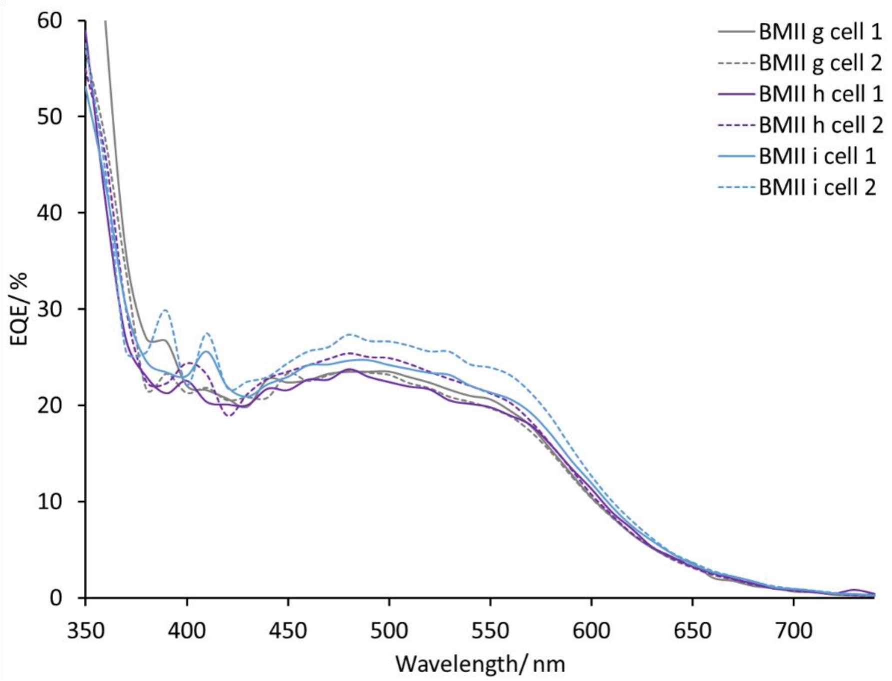

Fig. S6. EQE spectra for multiple DSCs with electrolytes BMIIg, BMIIh and BMIIi.

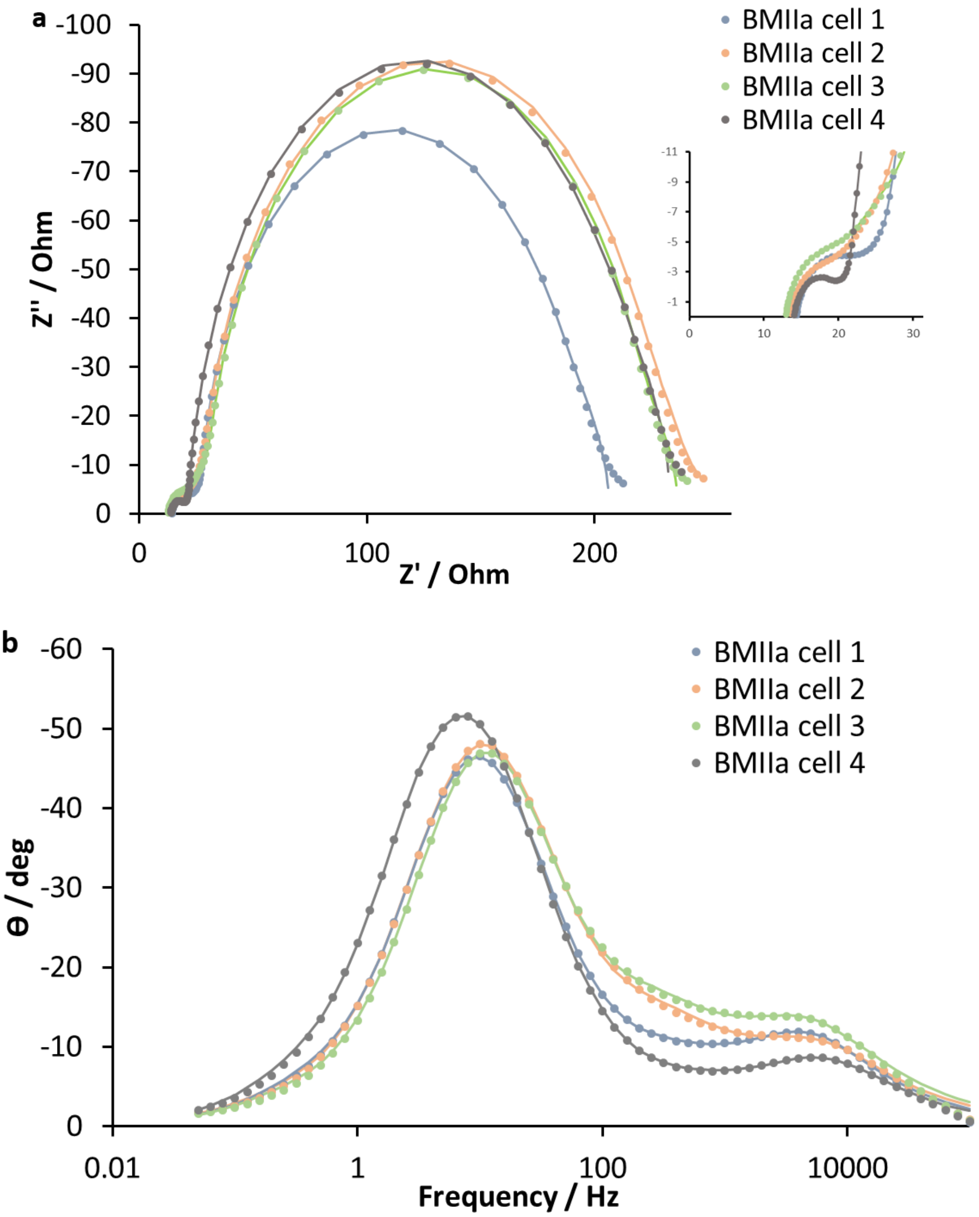

Fig. S7. EIS data of the multiple DSCs with electrolyte BMIIa. Solid lines represent fitted curves, dotted lines represent experimental data. (a) Nyquist plots, the expansion shows the high frequency region. (b) Bode plot

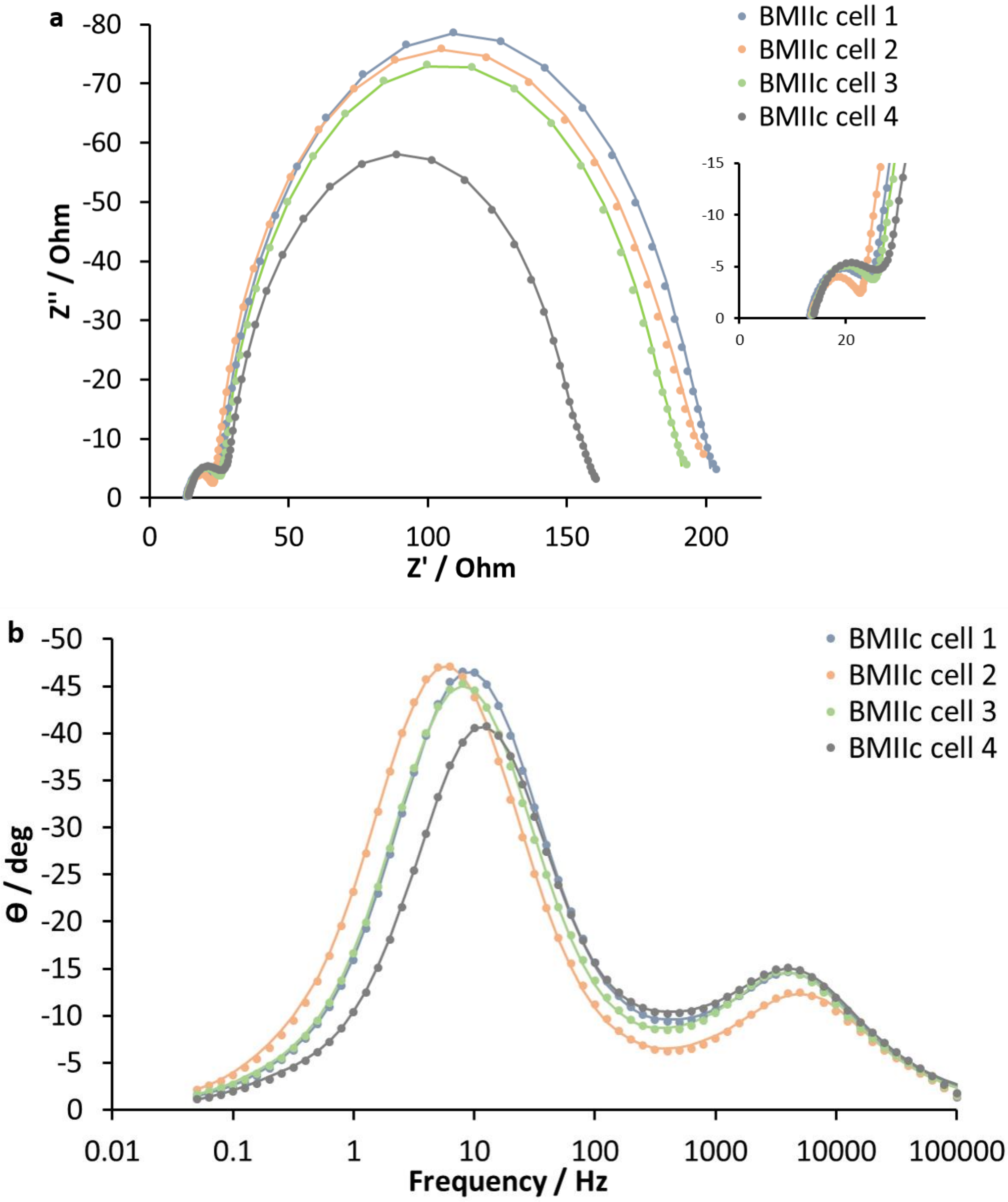

Fig. S8. EIS data of the multiple DSCs with electrolyte BMIIc. Solid lines represent fitted curves, dotted lines represent experimental data. (a) Nyquist plots, the expansion shows the high frequency region. (b) Bode plot.

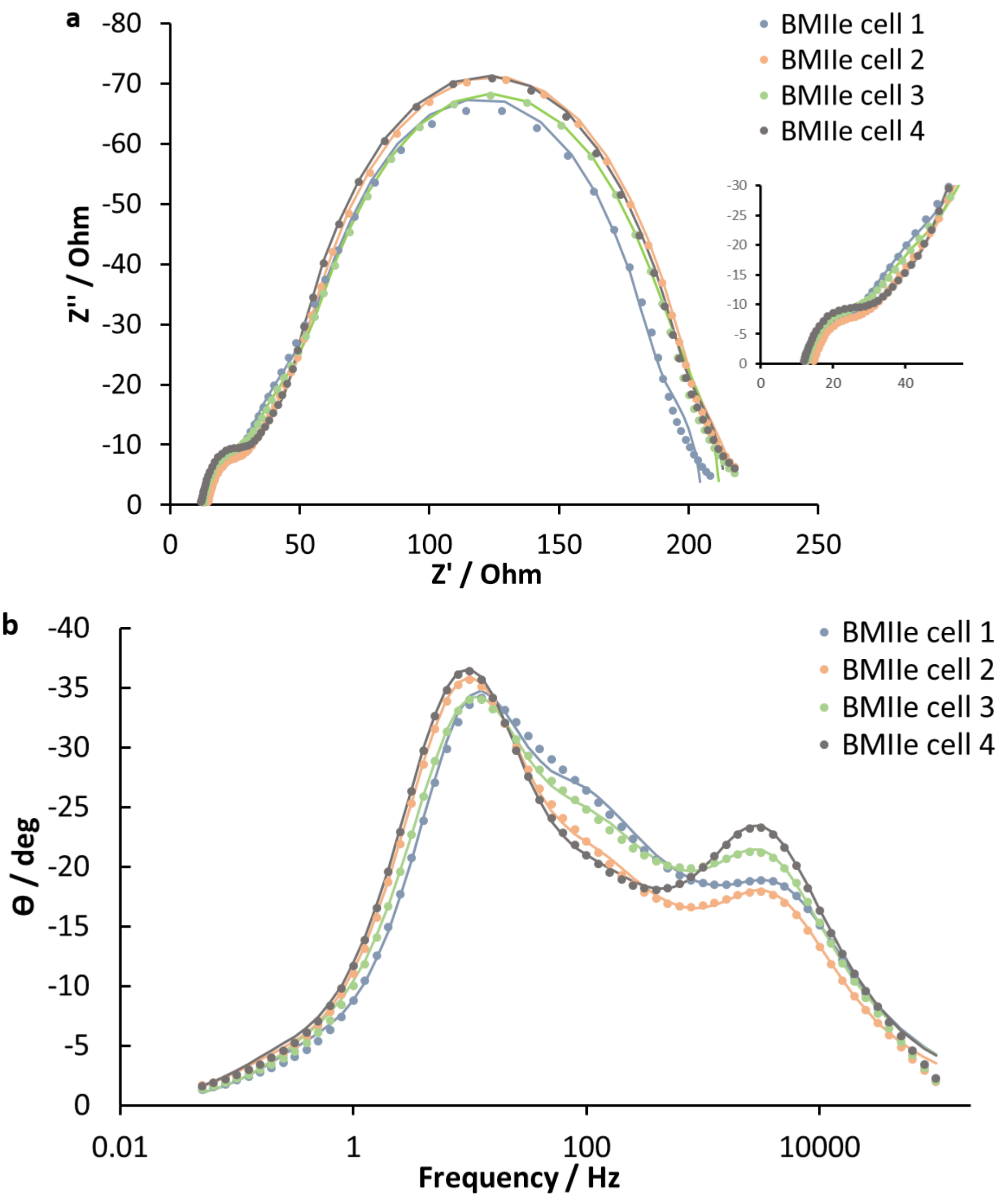

Fig. S9. EIS data of the multiple DSCs with electrolyte BMIIe. Solid lines represent fitted curves, dotted lines represent experimental data. (a) Nyquist plots, the expansion shows the high frequency region. (b) Bode plot.

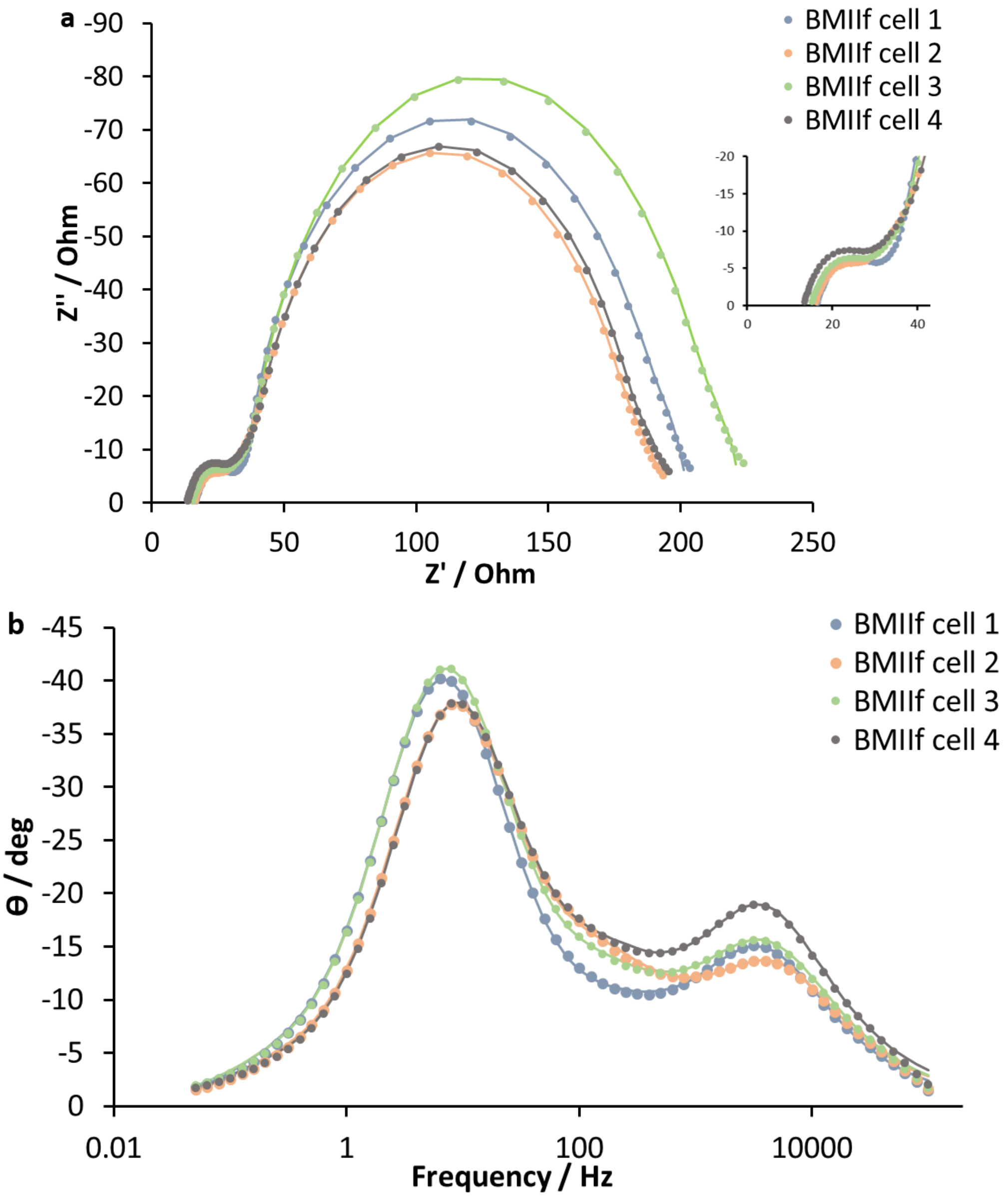

Fig. S10. EIS data of the multiple DSCs with electrolyte BMIIf. Solid lines represent fitted curves, dotted lines represent experimental data. (a) Nyquist plots, the expansion shows the high frequency region. (b) Bode plot.

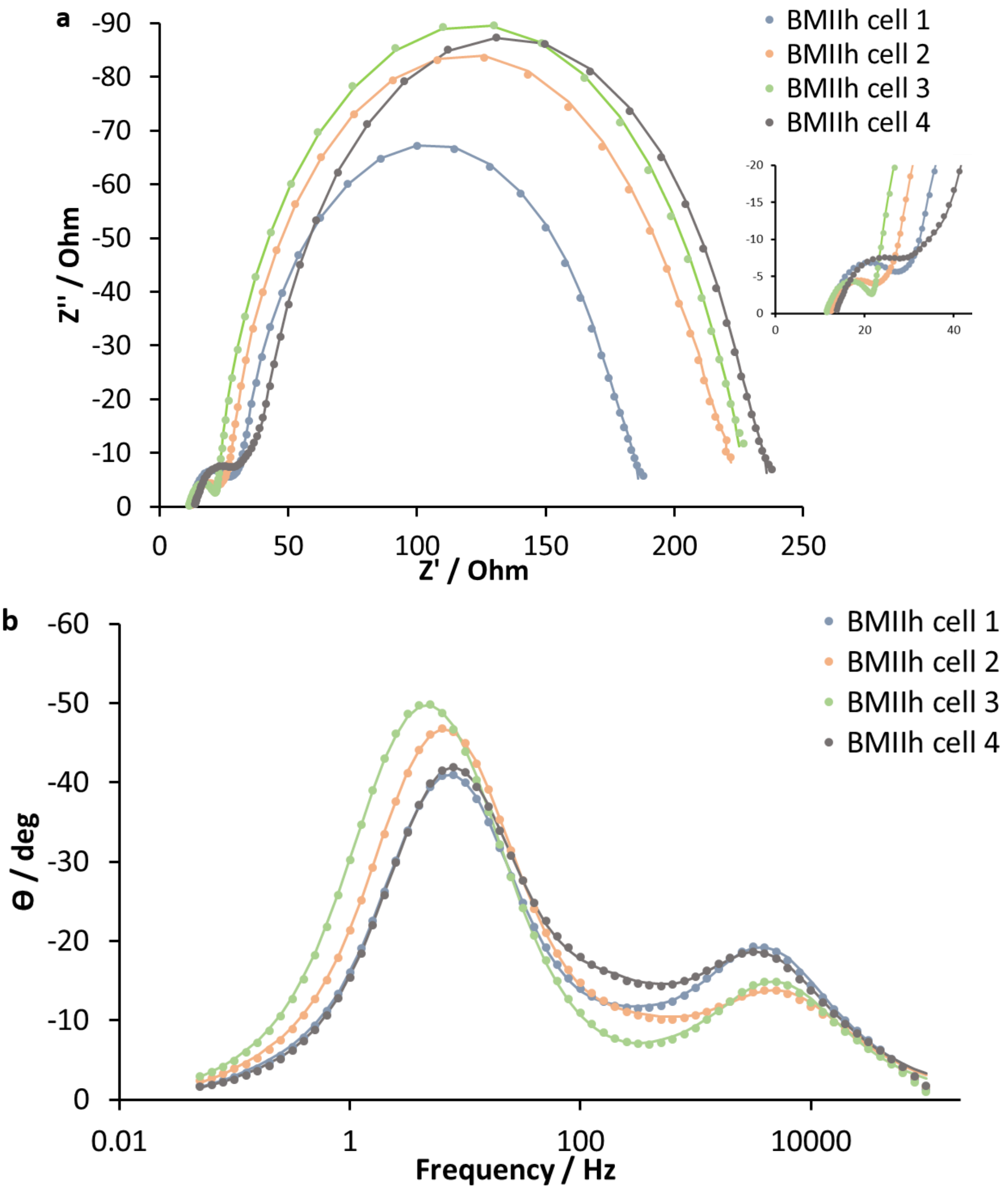

Fig. S11. EIS data of the multiple DSCs with electrolyte BMIIh. Solid lines represent fitted curves, dotted lines represent experimental data. (a) Nyquist plots, the expansion shows the high frequency region. (b) Bode plot.

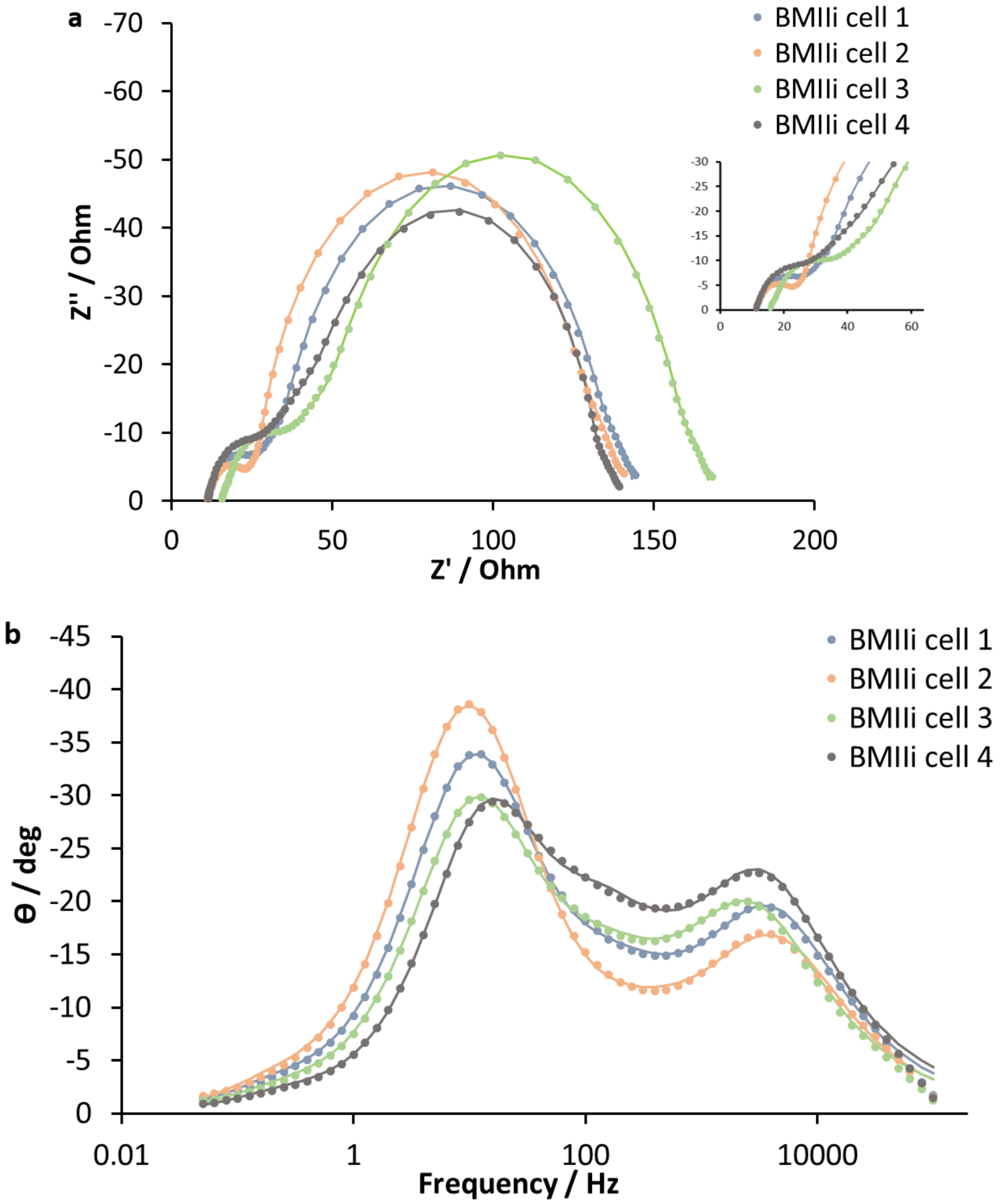

Fig. S12. EIS data of the multiple DSCs with electrolyte BMIIi. Solid lines represent fitted curves, dotted lines represent experimental data. (a) Nyquist plots, the expansion shows the high frequency region. (b) Bode plot.

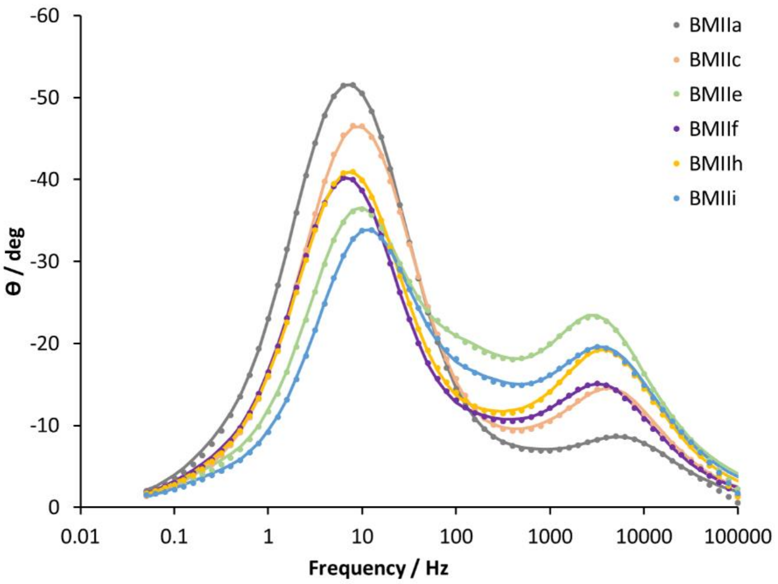

Fig. S13. Bode plot of the DSCs with electrolytes BMIIa, BMIIc, BMIIe, BMIIf, BMIIh, BMIIi. Solid lines represent fitted curves, dotted lines represent experimental data.

Table S2. EIS parameters for multiple DSCs using electrolytes with different concentrations of LiI.

Electrolyte	R_{rec} / Ω	C_{μ} / µF	R_{tr} / Ω	τ / ms	τ_t / ms	Ld / µm	R_s / Ω	R_{Pt} / Ω	C_{Pt} / µF
BMIIa cell 1	161	288	19	47	5	35	14	6	7
BMIIa cell 2	199	233	32	46	7	30	13	3	9
BMIIa cell 3	191	214	38	41	8	27	12	5	9
BMIIa cell 4	192	384	9	74	3	56	14	4	6
BMIIc cell 1	169	307	13	52	4	43	13	8	6
BMIIc cell 2	160	506	6	81	3	63	14	8	5
BMIIc cell 3	157	352	11	55	4	46	14	9	5
BMIIc cell 4	122	267	17	33	5	32	14	9	5
BMIIe cell 1	130	214	109	28	23	13	12	6	7
BMIIe cell 2	151	245	80	37	20	16	14	8	7
BMIIe cell 3	141	226	101	32	23	14	12	7	9
BMIIe cell 4	150	258	69	39	18	18	11	11	7
BMIIf cell 1	152	374	26	57	10	29	16	11	5
BMIIf cell 2	140	307	45	43	14	21	16	7	6
BMIIf cell 3	169	328	38	56	12	25	15	10	6
BMIIf cell 4	142	292	45	41	13	21	13	11	6
BMIIh cell 1	144	378	23	54	9	30	12	12	5
BMIIh cell 2	180	403	19	72	8	36	12	8	8
BMIIh cell 3	197	590	7	116	4	65	12	8	5
BMIIh cell 4	187	279	46	52	13	24	14	10	6
BMIIi cell 1	99	302	40	30	12	19	11	10	6
BMIIi cell 2	103	369	19	38	7	28	11	9	6
BMIIi cell 3	108	241	66	26	16	15	16	13	7
BMIIi cell 4	86	223	76	19	17	13	11	10	7

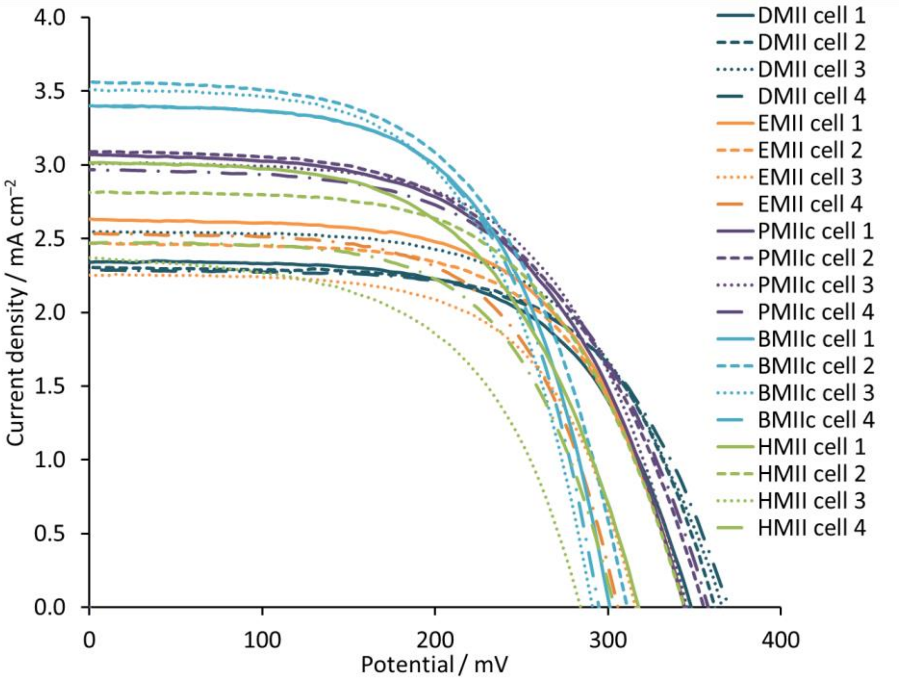

Fig. S14. J-V curves for the multiple DSCs with electrolytes DMII, EMII, PMIIc, BMIIc and HMII.

Table S3. Parameters for multiple DSCs using electrolytes based on imidazolium ionic liquids with different lengths of side-chain.

**Electrolyte**	J_{SC} / mA cm^{-2}	V_{OC} / mV	*ff* / %	*η* / %	**Rel.** *η* / %
DMII cell 1	2.31	362	63	0.53	9.5
DMII cell 2	2.35	348	62	0.50	8.9
DMII cell 3	2.55	366	60	0.56	10.0
DMII cell 4	2.29	370	62	0.52	9.3
EMII c ell 1	2.47	344	62	0.52	9.3
EMII c ell 2	2.63	344	61	0.55	9.8
EMII c ell 3	2.25	316	62	0.44	7.9
EMII c ell 4	2.53	306	62	0.48	8.6
PMIIc cell 1	3.01	315	62	0.59	10.5
PMIIc cell 2	2.98	270	62	0.50	8.9
PMIIc cell 3	2.98	318	63	0.59	10.5
PMIIc cell 4	-	-	-	-	-
BMIIc cell 1	3.40	301	59	0.61	10.9
BMIIc cell 2	3.56	311	57	0.63	11.3
BMIIc cell 3	3.51	291	58	0.59	10.5
BMIIc cell 4	3.40	294	60	0.60	10.7
HMII cell 1	3.14	316	60	0.60	10.7
HMII cell 2	3.00	317	62	0.59	10.5
HMII cell 3	3.05	333	62	0.63	11.3
HMII cell 4	3.13	317	61	0.61	10.9
N719	12.53	654	68	5.60	100

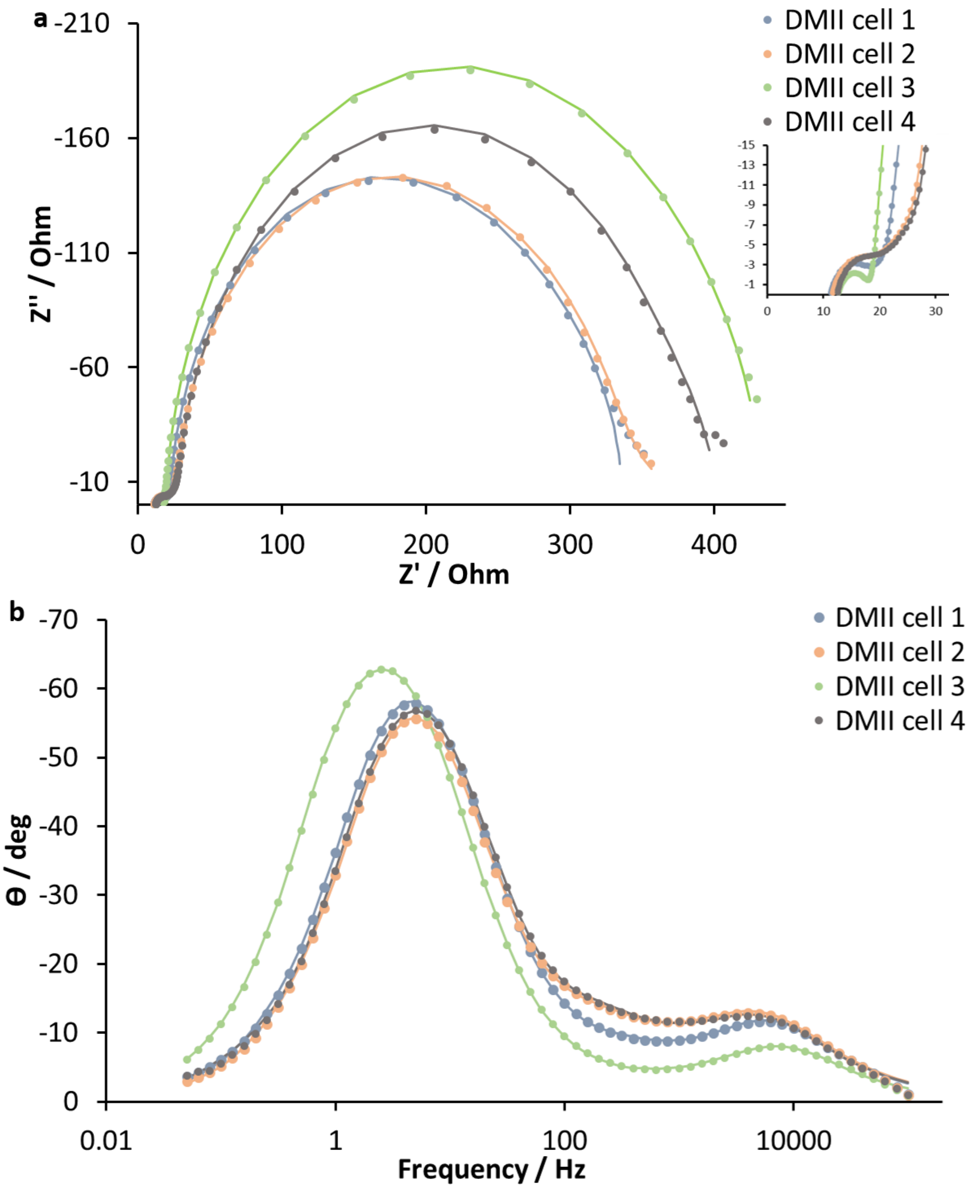

Fig. S15. EIS data of the multiple DSCs with electrolyte DMII. Solid lines represent fitted curves, dotted lines represent experimental data. (a) Nyquist plots, the expansion shows the high frequency region. (b) Bode plot.

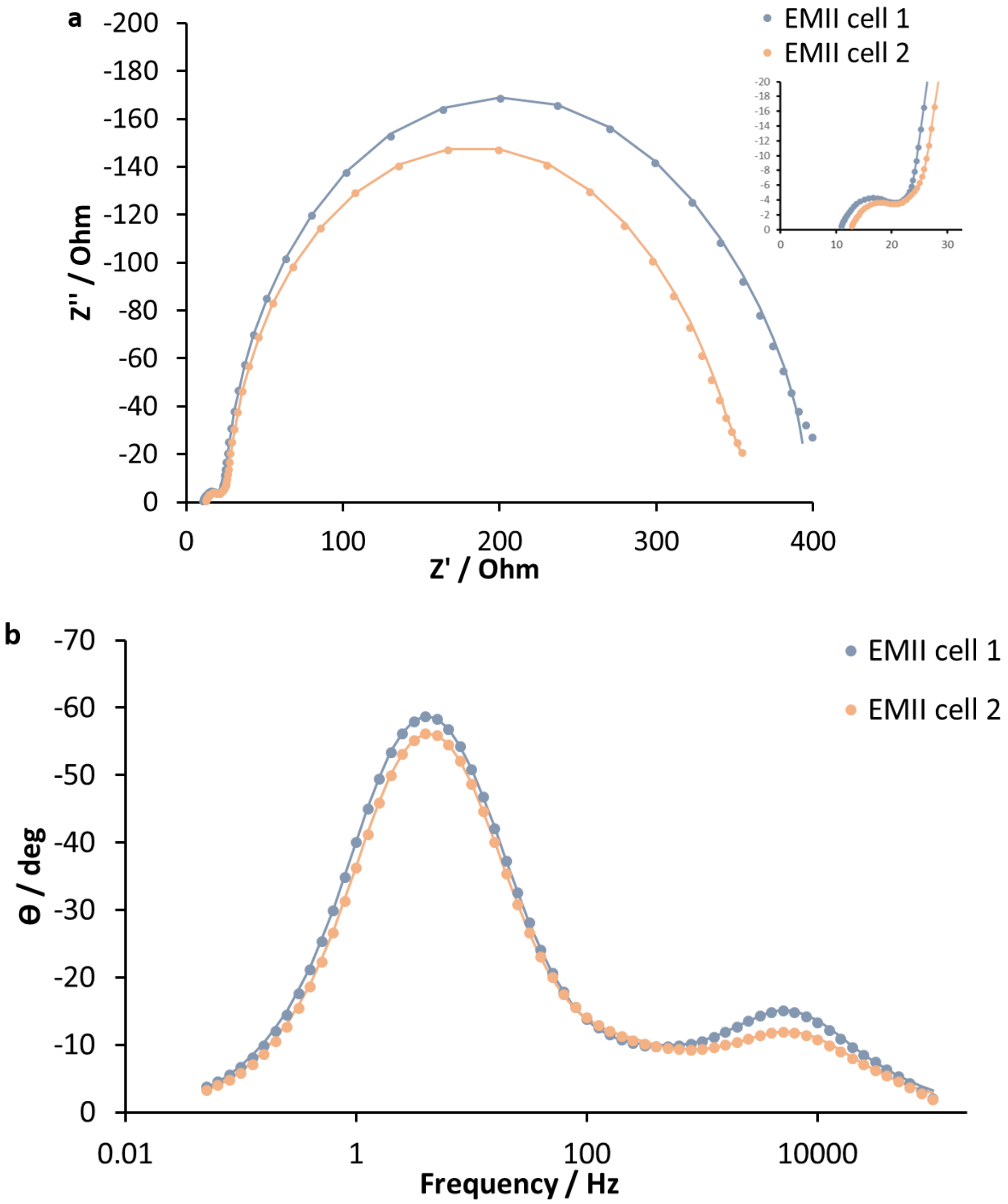

Fig. S16. EIS data of the multiple DSCs with electrolyte EMII. Solid lines represent fitted curves, dotted lines represent experimental data. (a) Nyquist plots, the expansion shows the high frequency region. (b) Bode plot.

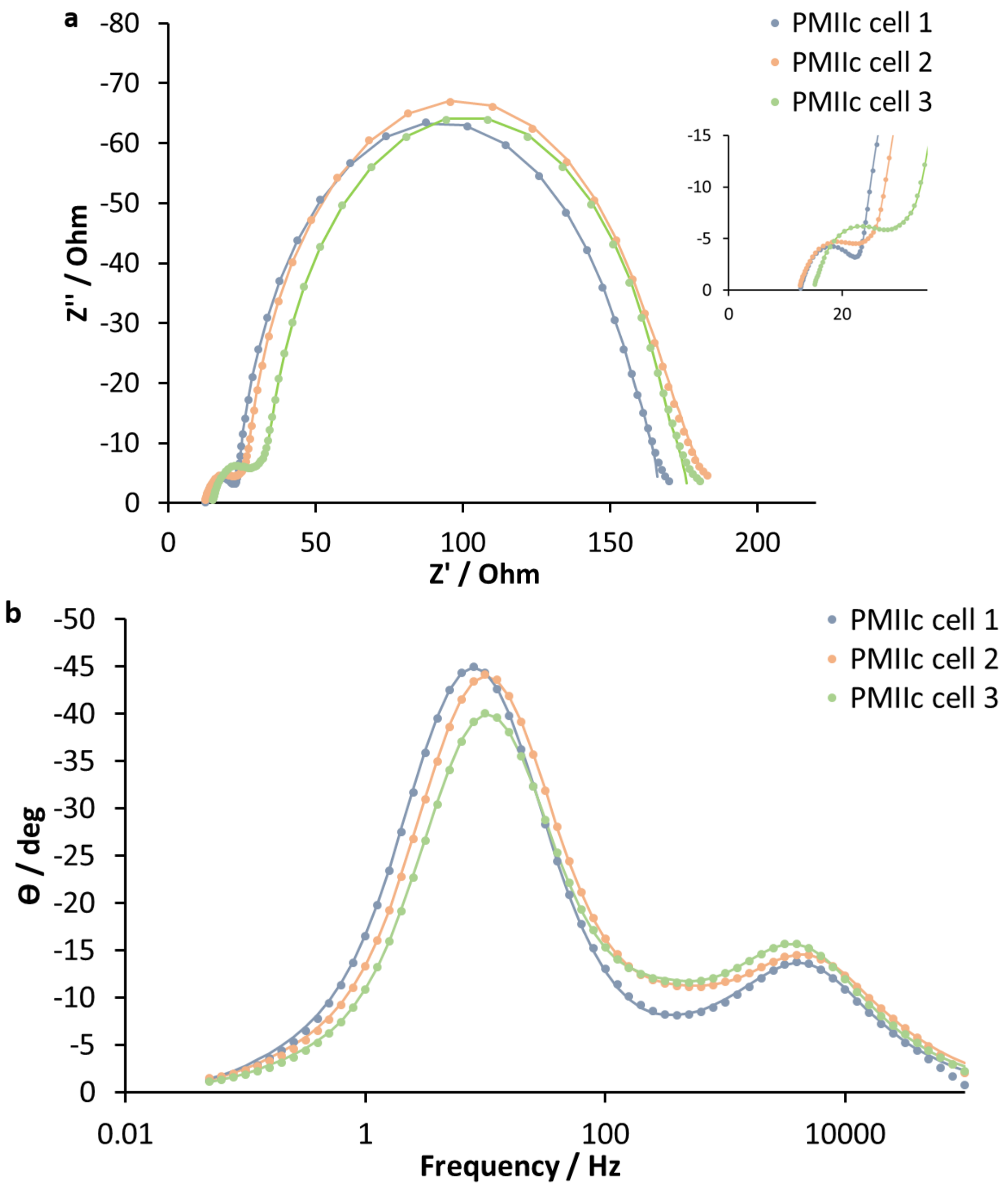

Fig. S17. EIS data of the multiple DSCs with electrolyte PMIIc. Solid lines represent fitted curves, dotted lines represent experimental data. (a) Nyquist plots, the expansion shows the high frequency region. (b) Bode plot.

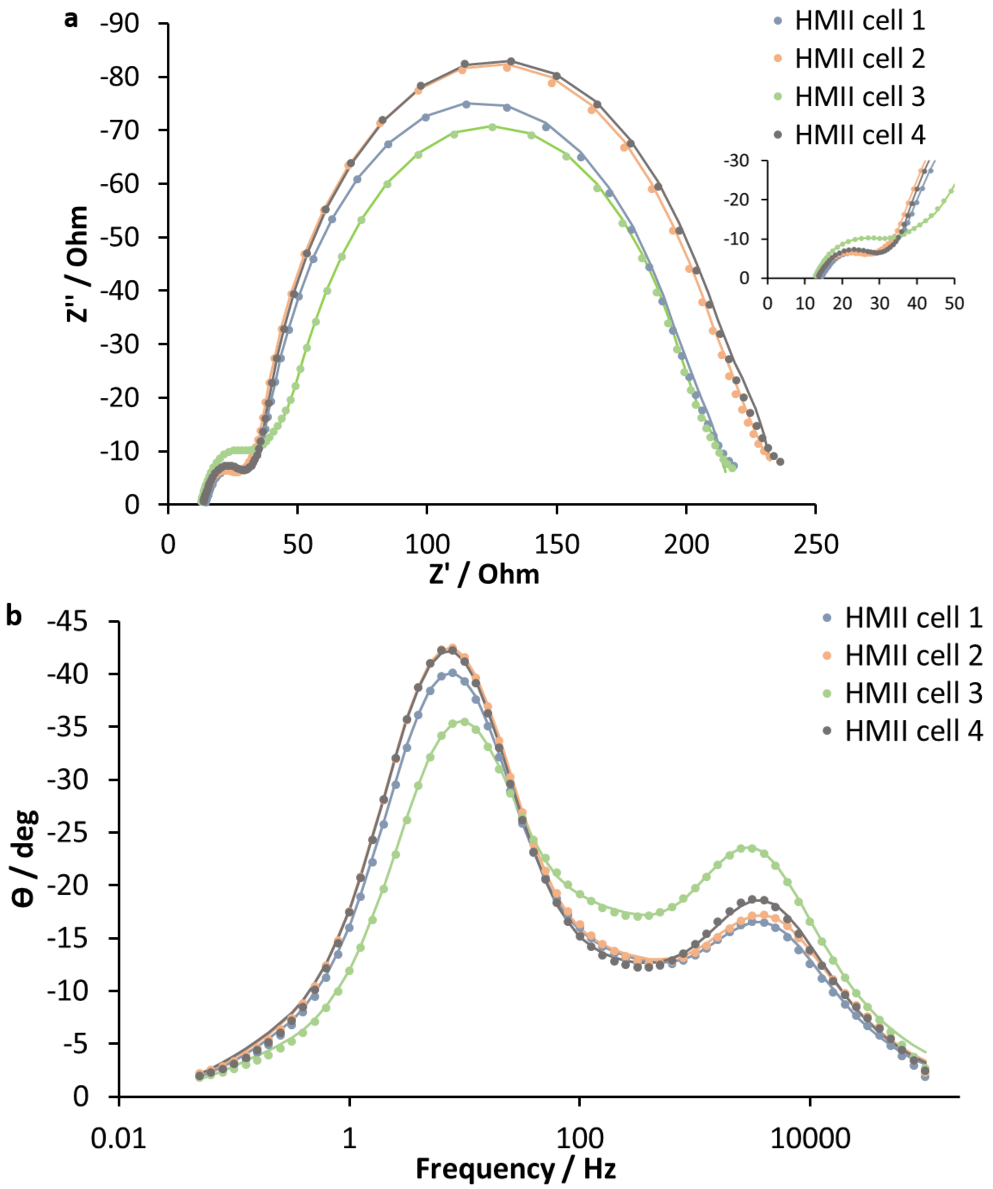

Fig. S18. EIS data of the multiple DSCs with electrolyte HMII. Solid lines represent fitted curves, dotted lines represent experimental data. (a) Nyquist plots, the expansion shows the high frequency region. (b) Bode plot.

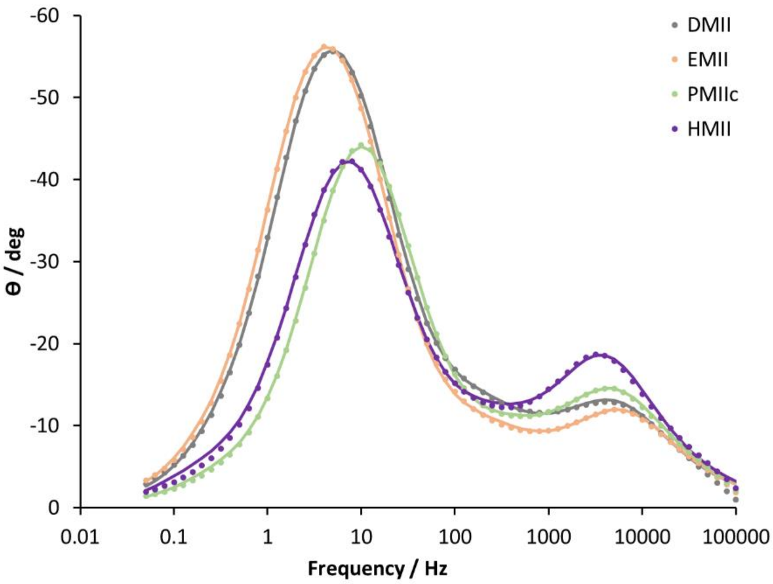

Fig. S19. Bode plot of the DSCs with electrolytes BMIIa, BMIIc, BMIIe, BMIIf, BMIIh, BMIIi. Solid lines represent fitted curves, dotted lines represent experimental data.

Table S4. EIS parameters for multiple DSCs using electrolytes based on imidazolium ionic liquids with different lengths of side-chain.

Electrolyte	R_{rec} / Ω	C_{μ} / µF	R_{tr} / Ω	τ / ms	τ_t / ms	Ld / µm	R_s / Ω	R_{Pt} / Ω	C_{Pt} / µF
DMII cell 1	266	484	10	129	5	61	11	6	6
DMII cell 2	269	416	22	112	9	42	12	6	7
DMII cell 3	381	768	4	293	3	122	13	4	6
DMII cell 4	337	363	26	122	9	43	12	5	7
EMII c ell 1	331	472	12	156	6	62	11	8	5
EMII c ell 2	299	464	19	139	9	48	13	6	5
PMIIc cell 1	134.00	405.35	8.37	54.35	3.39	48.01	12.64	7.90	6.05
PMIIc cell 2	140.70	297.41	18.99	41.84	5.64	32.66	12.33	7.75	5.45
PMIIc cell 3	134.30	265.95	26.49	35.72	7.04	27.02	14.93	9.76	6.97
HMII cell 1	164	329	35	54	12	26	14	10	5
HMII cell 2	179	330	32	59	11	28	13	10	5
HMII cell 3	155	256	62	40	16	19	12	13	6
HMII cell 4	179	328	30	59	10	29	13	11	5

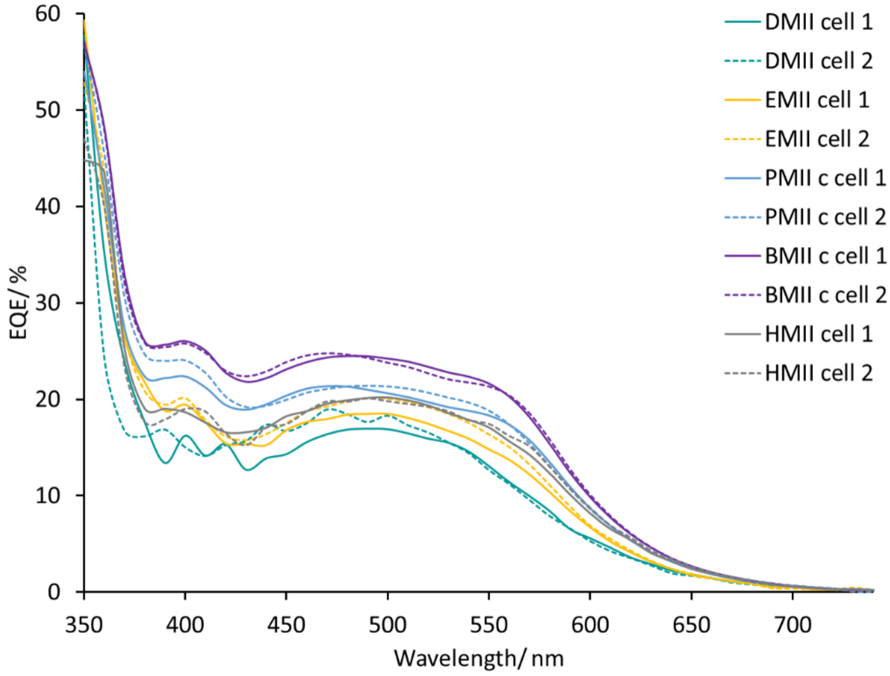

Fig. S20. EQE spectra for multiple DSCs using electrolytes DMII, EMII, PMIIc, BMIIc and HMII.

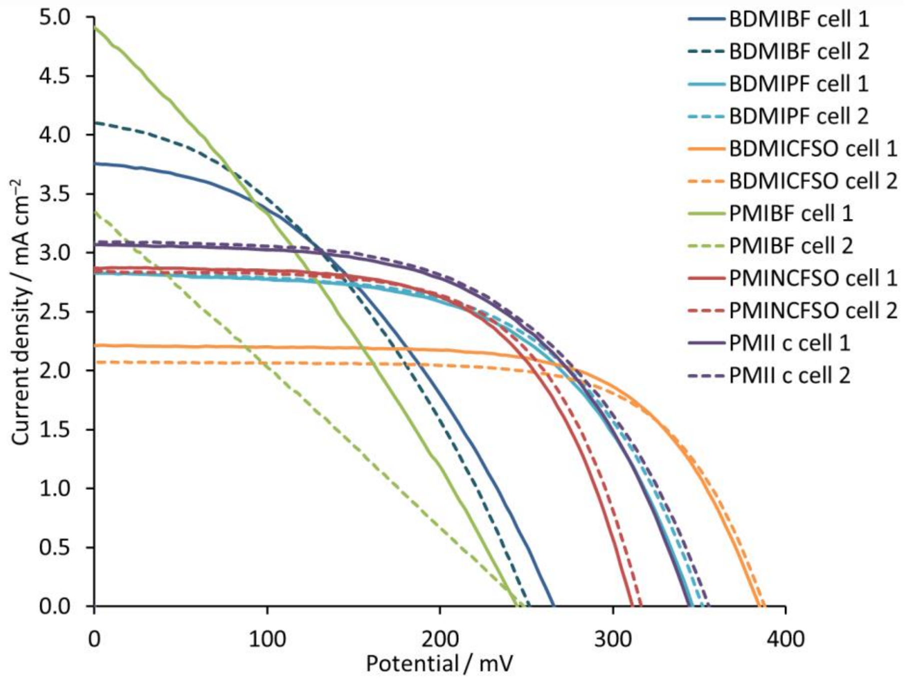

Fig. S21. J-V curves for the multiple DSCs with electrolytes based on imidazolium ionic liquids with different counter ions.

Table S5. Parameters for multiple DSCs using electrolytes based on imidazolium ionic liquids with different counter ions.

**Electrolyte**	**J_{SC} / mA cm^{-2}**	**V_{OC} / mV**	***ff* / %**	**η / %**	**Rel. η / %**
BDMIBF cell 1	3.80	266	41	0.41	7.3
BDMIBF cell 2	4.09	251	39	0.40	7.1
BDMIPF cell 1	2.84	351	58	0.57	10.2
BDMIPF cell 2	2.83	346	57	0.56	10.0
BDMICFSO cell 1	2.22	385	66	0.56	10.0
BDMICFSO cell 2	2.07	387	67	0.54	9.6
PMIBF cell 1	4.90	244	30	0.35	6.3
PMIBF cell 2	3.34	248	26	0.21	3.8
PMINCFSO cell 1	2.84	316	62	0.56	10.0
PMINCFSO cell 2	2.88	311	61	0.55	9.8
N719	12.53	654	68	5.60	100
